# Green synthesis of Carbonized Chitosan-Fe_3_O_4_-SiO_2_ nano-composite for adsorption of heavy metals from aqueous solutions

**DOI:** 10.1186/s13065-024-01257-5

**Published:** 2024-08-08

**Authors:** Dalia Amer Ali, Rinad Galal Ali

**Affiliations:** Department of Chemical Engineering, The British University in Egypt, El-Sherouk City, 11837 Egypt

**Keywords:** Adsorption, Water pollution, Copper, Nickel, Cobalt, Chitosan, Silicon dioxide

## Abstract

Water pollution with heavy metals owing to industrial and agricultural activities have become a critical dilemma to humans, plants as well as the marine environment. Therefore, it is of great importance that the carcinogenic heavy metals present in wastewater to be eliminated through designing treatment technologies that can remove multiple pollutants. A novel green magnetic nano-composite called (Carbonized Chitosan-Fe_3_O_4_-SiO_2_) was synthesized using Co-precipitation method to adsorb a mixture of heavy metal ions included; cobalt (Co^2+^), nickel (Ni^2+^) and copper (Cu^2+^) ions from aqueous solutions. The novelty of this study was the synthesis of a new nano-composite which was green with magnetic properties to be more sustainable and environmentally friendly. Its magnetic properties made it separated easily from solutions after accomplishment of the adsorption process using a magnet. Extended Freundlich isotherm was the best fitted model with maximum adsorption capacity of the metal ions mixture 2908.92 mg/g. Different experimental parameters have been studied included the initial concentration for a mixture of nickel, cobalt and copper metal ions (0.05–0.1 molar), dosage of adsorbent (0.5–3.5 g/L) and contact time (6–90 min) to investigate their changing effect on the removal percents of the heavy metal ions mixture from aqueous solutions. The experimental adsorption percent of cobalt ion ranged from 1.58 to 64.28%, nickel ion adsorption percent ranged from 10.68 to 94.12% and copper ion adsorption percent ranged from 4.41 to 76.23% at pH = 9 were based on the combination of the adsorption process parameters.

## Introduction

Over the last few years, removing heavy metal ions from aqueous solutions has become increasingly important, whether as a means of pollution control or for the recovery of raw materials [1, 2]. Heavy metal contamination in humans affects numerous organs, including the kidneys, the liver, the stomach, the mental environment, and the reproductive system [3]. Humans are also susceptible to carcinogenic effects caused by heavy metals [3]. Nickel (Ni), cobalt (Co) and copper (Cu) are most popular toxic heavy metals [4]. These heavy metals can still be hazardous even when they are detected in traces [4]. Nickel exists in the effluent wastewater from stainless steel and nickel alloy production [4]. While cobalt resources are paint, pigment and electroplating industries [5]. The copper main resources are corroded plumbing systems, electronic and cables industry [2]. The maximum permissible levels of nickel, cobalt and copper in drinking water are 0.07 mg/l, 0.05 mg/L, 2 mg/L, respectively, according to the world health organization (WHO) recommendations [6, 7]. Nanotechnology is defined as the design and fabrication of materials, devices and systems with control at nano-meters dimensions [8]. The essence of nanotechnology is therefore size and control [9]. Nanotechnology is considered nowadays as a promising alternative technology for removal of various contaminants from wastewater [10]. Nano-adsorption and NF are considered to be promising techniques introduced by nanotechnology for wastewater treatment [10].

Chemical precipitation, biological methods, ion exchange, and adsorption are some of the methods for removing heavy metal ions from aqueous solutions [8, 9]. Chemical precipitation, also defined as coagulation precipitation, is a widely employed technique in industry and is regarded as influential and mature [2]. Biotechnological methods use the natural characteristics of microorganisms to soak up and collect heavy metals [11]. Biological method is a more complex and costly method of heavy metal elimination from wastewater due to production of large amounts of biomass [12]. Adsorption is widely used in heavy metals removal from wastewater due to the numerous advantages it offers [2]. It has various benefits over different forms of contaminant reduction for it is easy to develop and execute technologically easy and is flexible to multiple treatment forms. It operates in favorable working circumstances and a broad pH range [13]. Nano-adsorbents are better than the conventional adsorbents because, they have larger surface areas and more active sites which give them higher degree of pollutants selectivity [10]. The carbon-based adsorption technique is widely applied in environmental protection, and biomass-derived carbon is recognized as one of the most economical and promising adsorbents for heavy metal removal due to its high porosity and thus high adsorption capacity [14]. In addition, nano-silica is considered to be mineral adsorbent that has a high adsorption capacity for heavy metal ions at low operating costs [13]. The presence of hydroxyl, carboxyl and amino groups on the surface of adsorbent increases its affinity to adsorb heavy metals from wastewater [15, 16]. According to the previous studies, many adsorbents removed low concentrations of heavy metals from aqueous solutions [2]. Therefore, it becomes necessary to create and improve the properties of adsorbents to increase their ability in removal of high concentrations of heavy metals from aqueous solutions. Thus, the first objective of this research study is to create a novel green magnetic nano-composite consists of (Carbonized Chitosan) as a precursor material which will be functionalized with magnetite (Fe_3_O_4_) and silica (SiO_2_) extracted from a bio-waste (sugarcane bagasse) to increase its adsorption capacity towards high concentrations of heavy metals. The functionalization of the (Carbonized Chitosan) with magnetite and silica will be performed in this study in order to combine the benefits of high porosity and stability of the (Carbonized Chitosan) with high capacity of silica towards heavy metals and the magnetic properties of magnetite that will facilitate the separation of the new nano-composite after adsorption process. A second objective of this study is to evaluate the ability of this synthesized novel green nano-composite in simultaneous removal of high concentration mixtures of heavy metal ions (Co^2+^), (Ni^2+^) and (Cu^2+^) from aqueous solutions.

## Materials and methods

### Chemicals

Sugarcane bagasse was collected from a farm at Alsharkeya governorate, Egypt. All chemicals used in this study were analytical grade reagents including; Ferric chloride hexahydrate (FeCl_3_.6H_2_O), Ferrous sulphate heptahydrate (FeSO_4_·7H_2_O), Copper sulphate pentahydrate (CuSO_4_.5H_2_O), Cobalt (II) chloride (COCL_2_), Nickel (II) chloride hexahydrate (NiCl_2_.6H_2_O) were provided by Sigma Aldrich Company. Chitosan (C_6_H_11_NO_4_)_n_, Sodium hydroxide (NaOH) and Hydrochloric acid (HCl 36% v/v) were provided by Nano-Gate Company, Egypt. Deionized water was used for preparation of all solutions.

### Equipment

The morphology of the extracted sodium silicate (Na_2_SiO_3_) and the (Carbonized Chitosan-Fe_3_O_4_-SiO_2_) were conducted by scanning electron microscope (SEM, Quattro s – Thermo Scientific, Netherland) and Transmission Electron Microscope (TEM) (JEM-1400Flash, JEOL Solutions for Innovation Company, USA). The specific surface area, pore-size and particle size were determined by Brunauer-Emmett-Teller (BET) (NOVA touch, Quantachrome Company, U.S.A). The purity of the prepared nano-composite was conducted by X-Ray Diffraction (XRD) (Empyrean – Malvern Analytical Company, Netherland). The identification of the groups existed on the surface of the synthesized nano-composite was investigated using Fourier Transformation Infrared (FTIR) spectra (Vertex 70 RAM II, Germany). The surface charge of the new prepared nano-composite was determined using dynamic light scattering instrument (ZetaSizer Nano Series (HT), Nano ZS, Malvern Instruments, UK). Filtration of sodium silicate (Na_2_SiO_3_) was performed using vacuum filtration apparatus (RS-1, Shenzhen Educational Equipment, China). Vacuum dryer (Thermo-Fisher Scientific Company, USA) was used for drying the final product.

### Kinetics models

The rate of acceptance of the dissolve and the period required for adsorption are both heavily influenced by adsorption kinetics [17]. It is employed to evaluate the effectiveness of the adsorbent and its mass transfer processes. Pseudo First Order (PFO) and Pseudo Second Order (PSO) kinetic models were used to study the kinetics at various time points for adsorbing the mixture of Co^2+^, Ni^2+^ and Cu^2+^ metal ions [17]. The kinetic models of PFO and PSO were characterized by Eqs. (1) and (2) [18].

1 $$\\\text{PFO}:\text{L}\text{o}\text{g}\:\left(\text{q}\text{e}-\text{q}\right)=\:\text{L}\text{o}\text{g}\:\left(\text{q}\text{e}\right)-\frac{\text{k}1\text{*}\text{t}}{2.303}$$

2 $$PSO:\:\:\frac{t}{qt\:}=\:\frac{1}{k2*{qe}^{2}}+\left(\frac{1}{qe}\right)*t$$

Where q_t_ and q_e_ are, respectively, the quantity of heavy metal ions that the adsorbent has absorbed at equilibrium and time (t). The rate constants for PFO and PSO are k_1_ and k_2_, respectively

### Single-component isotherm models

The ratio between the equilibrium solute concentration on the exterior of the adsorbent (qe) and the contacting solute quantity in the liquid (Ce) are related by a curve known as the adsorption isotherm [19, 20]. It is typically used to analyze porous solids, design the adsorption procedure, and determine the adsorbent’s specific surface area [19, 20].

### Langmuir

The idea of monomolecular adsorption on a regular exterior is demonstrated by the Langmuir isotherm model. This model can be described by Eq. (3) [15, 16]:

3 $$\:\frac{Ce}{qe}=\left(\frac{1}{qm}\right)\text{*}Ce+\frac{1}{qm\text{*}KL}$$

The Langmuir constant, K_L_, represents the adsorption energy (L/mg), and q_m_ is the optimum monolayer adsorption capacity (mg/g). C_e_ and q_e_ are the concentrations at equilibrium of the heavy metal ions mixture in (mg/L) in the solution and solid phases, respectively.

### Freundlich

The adsorption characteristics of the heterogeneous surface are revealed by this model [19]. The two-parameter Freundlich isotherm model is described by the following Eq. (4) [21, 22]:

4 $$\:Log\:qe=Log\:{K}_{H}+\frac{1}{n}*Log\:Ce$$

Where n and K_H_ (L/mg) are the Freundlich constants, which represent, respectively, adsorption intensity and capacity.

#### Dubinin-Radushkevich

The process of adsorption onto heterogeneous surfaces is represented in this model by a Gaussian energy distribution [21, 22]. This model, which is temperature-dependent, can be used for physical adsorption. Only pollutants with intermediate concentration ranges can be employed with this model [21, 22]. Following are the Eqs. (5) and (6) that represent the Dubinin-Radushkevich isotherm model [21, 22]:

5 $$\:\text{L}\text{n}\:\text{q}=Ln\:qmax-\:\beta\:{R}^{2}{T}^{2}{Ln}^{2}(1+\:\frac{1}{C})$$

6 $$\:\text{E}=\frac{1}{\sqrt{2\beta\:}}$$

Where $\:\beta\:$ (mol^2^/kJ^2^) denotes an adsorption energy constant, q_max_ (mg/g) denotes the optimum adsorption capacity, and E denotes the free energy/adsorbate molecule (kJ). According to [22, 24] the quantity of E is utilized to tell the different kinds of adsorption approaches apart:

- A physical adsorption will occur whenever free energy/adsorbate molecule (E) is < 8 kJ/mol.
- If 8 kJ/mol < E < than 16 kJ/mol, then ion exchange or chemical adsorption will take place.
- The reaction will be governed by particle diffusion if E > 16 kJ/mol.

### Multiple-component isotherm models

The multi-component adsorption idea is now crucial to wastewater treatment procedures since wastewater today contains significant quantities of multiple contaminants. This is because single-component isotherm models are unable to capture the aggressive interchanges among the contaminants current in the contaminated water. Additionally, the entire adsorption process is significantly impacted by the competitive interactions between pollutant molecules. To demonstrate multiple-component adsorption systems, Modified Langmuir isotherm models and Extended Freundlich isotherm can be employed [23].

### Extended freundlich

In the event that interactions between the molecules of deposited contaminants occur, this model is utilized to depict multilayer adsorption procedures on diverse exteriors [24, 25]. The following Eqs. (7) and (8) represent the extended Freundlich isotherm model [24, 25].

7 $$\:\text{q}\text{e},1=\frac{KF,1.\:\:{Ce,1}^{\left(\frac{1}{n1}\right)+X1}}{{Ce,1}^{X1}+y1.{Ce,2}^{Z1}}$$

8 $$\:\text{q}\text{e},2=\frac{KF,2.\:\:{Ce,2}^{\left(\frac{1}{n2}\right)+X2}}{{Ce,2}^{X2}+y2.{Ce,2}^{Z2}}$$

Where the equilibrium adsorption capacities for components 1 and 2 are respectively, q_e,1_ and q_e,2_ in (mg/g). The Freundlich constants for components 1 and 2 are K_F,1_ and K_F,2_, respectively. The equilibrium concentrations of components 1 and 2 are C_e,1_ and C_e,2_, respectively, in (mg/L). The adsorption strengths for elements 1 and 2 are given by the numbers n_1_ and n_2_, which are derived from experimental data for distinct Freundlich isotherms.

### Modified langmuir

This model clarifies how pollutant molecule interactions take place in a solution. In order to highlight the competing impact of the pollutant molecules in the solution and to understand the nature of the adsorption process, this model also incorporates the interaction factor [23, 25]. Equation (9) is used to represent the modified Langmuir isotherm model [23, 25].

9 $$\:\text{q}\text{e},\text{i}=\frac{qm,i\:.\:\:KL,i\:.\:\left(\frac{Ce,i}{\eta\:L,i}\right)}{1+{\sum\:}_{j=1}^{N}(KL,j\:.\:\left(\frac{Ce,j}{\eta\:L,j}\right))}$$

The Langmuir isotherm model illustrates the basic idea behind monomolecular adsorption on uniform surfaces. By applying the Langmuir constant for component i, K_L, i_ (L/mg), which is derived from experimental data of individual Langmuir isotherms, one may determine the adsorption capacity of component i, q_e, i_ (mg/g) at equilibrium. The calculation additionally takes into account the equilibrium concentration of component i, C_e, i_ (mg/L) and the monolayer adsorption capacity for component i, q_m, i_ (mg/g). The interaction factor L_,i_ is determined by the concentration of the other components, and N represents the total number of components in the solution. Using Microsoft Excel’s solver tool for non-linear regression, L_,i_ and L_,j_ were computed.

### Statistical error function

In order to compare the application of different multicomponent isothermal models through nonlinear regression using the least squares method, Marquardt standard deviation (MPSD) was used as a tool for determining the best fitting isothermal equation [25]. This can be calculated using the following Eq. (10) [25];

10 $$\:MPSD=\:100\sqrt{\frac{1}{n-p}*\sum\:_{i=1}^{n}{\left(\frac{{q}_{e,means}-\:{q}_{e,calc}}{{q}_{e,means}}\right)}^{2}}$$

Where; q_e, calc_ (mg/g) is the calculated adsorption capacity at equilibrium, q_e, mean_ (mg/g) is the average of q_e, exp_, (n) is the number of the experimental data and (p) is the number of factors in each isotherm model.

### Extraction of sodium silicate (Na_2_SiO_3_) from sugarcane bagasse

**Fig. 1:**
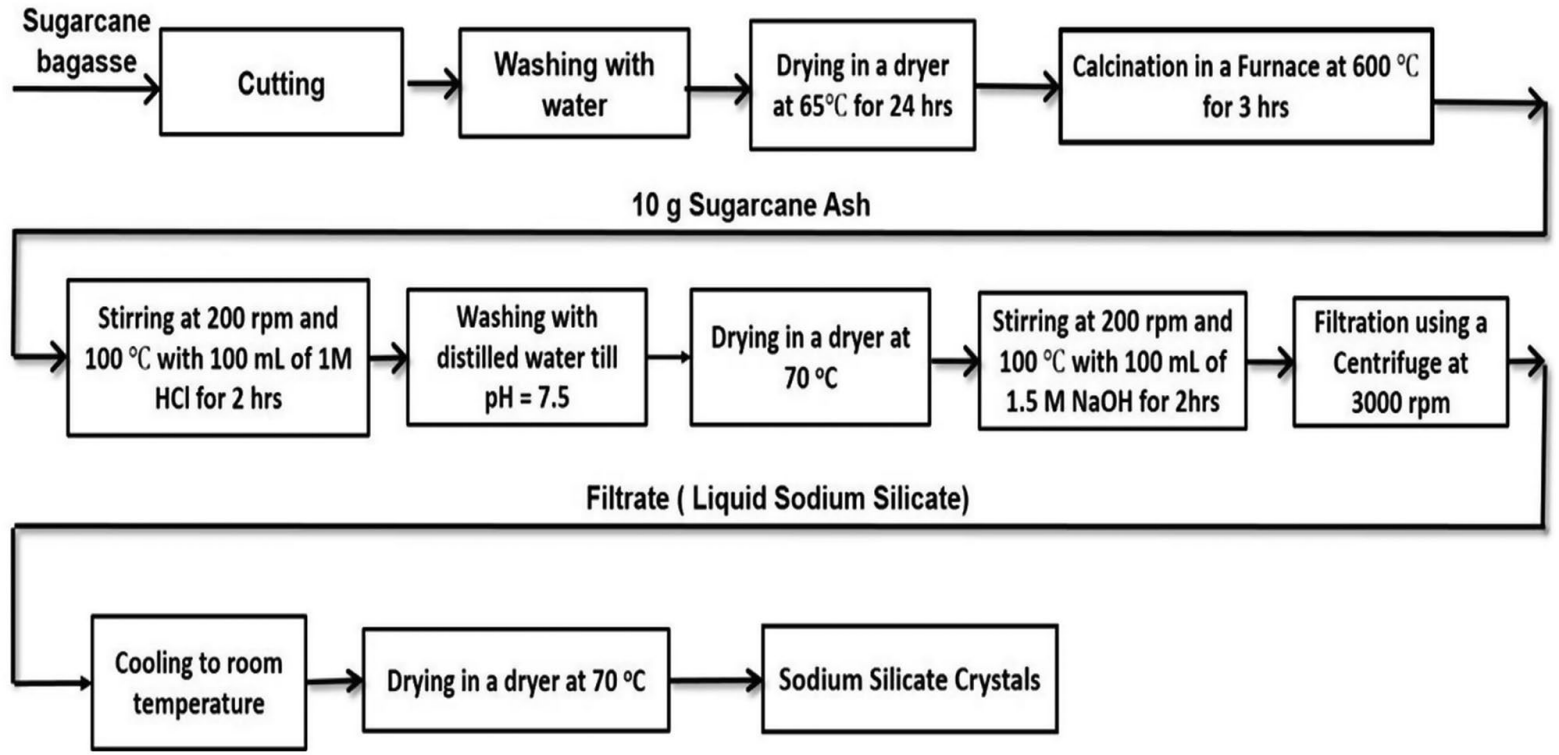
Extraction scheme of sodium silicate from sugarcane bagasse

As shown in Fig. 1, small pieces of sugarcane bagasse were washed with distilled water then dried for a day at 65 ^o^C. Calcination process at 600 ^o^C for 3 h was carried out for the dried sugarcane bagasse. The extraction of sodium silicate from the bagasse ash was performed in a two-neck round bottom flask with a condenser where 100 mL of 1 M HCl was mixed at 200 revolutions per minute (rpm) with 10 g of the bagasse ash under heating at 100 °C for 2 h to increase the weight% of SiO_2_ and to decrease the other mineral oxides. Followed by washing the precipitate with distilled water until reaching a neutral pH and drying at 70 °C in a dryer for 2 h. Then a solution of 100 mL NaOH (1.5 M) was mixed with the precipitate at 100 ^o^C for 2 h. Finally, a filtration of the resulted sodium silicate crystals was achieved using a centrifuge at 3000 rpm for 10 min, cooling to room temperature, drying at 70 ^o^C in a dryer and storing in a dessicator.

The following Eq. (11) represented the alkaline treatment of the bagasse ash using 1 M NaOH solution which reacted with silica (SiO_2_) to produce sodium silicate (Na_2_SiO_3_) and water (H_2_O) [26].

11 $${\rm{Si}}{{\rm{O}}_{\rm{2}}}{\rm{ + 2 NaOH\rightarrow N}}{{\rm{a}}_{\rm{2}}}{\rm{Si}}{{\rm{O}}_{\rm{3}}}{\rm{ + }}{{\rm{H}}_{\rm{2}}}{\rm{O}}$$

### Synthesis of (Carbonized Chitosan-Fe_3_O_4_-SiO_2_) green magnetic nano-composite

**Fig. 2:**
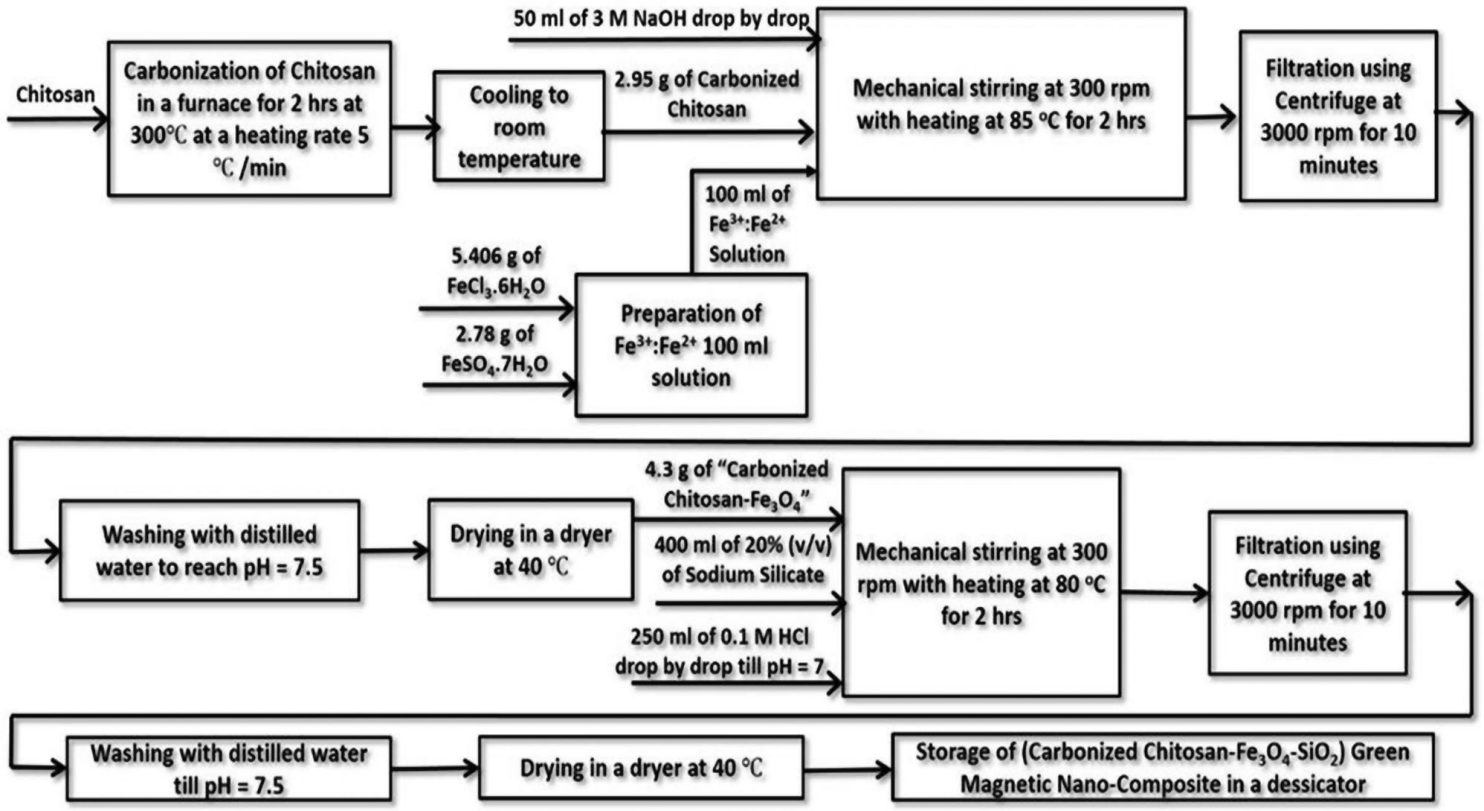
Preparation scheme of the (Carbonized Chitosan-Fe_3_O_4_-SiO_2_) green magnetic nano-composite

Since Carbonized Chitosan has a large specific surface area, it is used as the precursor for the composite. As represented in Fig. 2, Chitosan was carbonized in a furnace at 300$\:^\circ\:C$ and 5$\:^\circ\:C$/min for 2 h followed by cooling until room temperature was reached. For co-precipitation of Magnetite (Fe_3_O_4_) on the produced Carbonized Chitosan; 2.95 g of the Carbonized Chitosan was mixed with 100 ml solution of Fe^3+^: Fe^2+^ which was prepared in a molar ratio 2:1 using FeCl_3_.6H_2_O and FeSO_4_.7H_2_O as the precursor salts, respectively. 50 ml of 3 M NaOH was added to the previous mixture in a drop by drop wise with mechanical stirring at 300 rpm and heating at 85 $\:^\circ\:C$ for 2 h. Filtration of the produced composite (Carbonized Chitosan-Fe_3_O_4_) took place through centrifugation at 3000 rpm for 10 min, rinsing with distilled water until neutral pH then drying in a dryer at 40$\:^\circ\:C$. For co-precipitation of SiO_2_ on the produced composite (Carbonized Chitosan-Fe_3_O_4_); 400 ml of sodium silicate solution 20% (v/v) was mixed with 4.3 g of (Carbonized Chitosan-Fe_3_O_4_). 250 ml of 0.1 M HCl was incorporated to the previous mixture in a drop-by-drop wise till reaching neutral pH with mechanical stirring at 300 rpm and heating at 80$\:^\circ\:C$for 2 h. Filtration of the produced composite (Carbonized Chitosan-Fe_3_O_4_-SiO_2_) has been accomplished using a centrifuge at 3000 rpm for 10 min followed by washing with distilled water until reaching neutral pH then drying in a dryer at 40$\:^\circ\:C$and finally storage of the composite in a desiccator. The following reaction Eqs. (12) and (13) took place during the synthesis of the new nano-composite (Carbonized Chitosan-Fe_3_O_4_-SiO_2_):

### •Co-precipitation reaction for production of magnetite

12 $$\eqalign{& \>2FeC{l_3}{\mkern 1mu} {\mkern 1mu}\cdot 6{H_2}O + \cr & FeSo4{\mkern 1mu}\cdot 7{H_2}O + 8NaOH \to Fe3{O_4}\left( s \right) + \cr & N{a_2}S{O_4} + 6NaCl + 23{H_2}O \cr}$$

### •Sodium silicate reaction with hydrochloric acid to produce silica

13 $$N{a_2}Si{O_3} + {\rm{ }}2{\rm{ }}HCl \to Si{O_2} + {\rm{ }}2{\rm{ }}NaCl{\rm{ }} + {\rm{ }}{H_2}O$$

### Adsorption batch experiments

At pH = 9, adjusted with 0.1 M NaOH solution, heavy metals (Cu^2+^, Co^2+^, and Ni^2+^) were adsorbed from aqueous solutions. Using CuSO_4_.5H_2_O, COCl_2_ and NiCl_2_.6H_2_O, initial concentrations of heavy metals ion were prepared. The experiments were carried out in conical glass flasks that were shaken vigorously at 200 rpm using a laboratory shaker. After adsorption, each heavy metal ion concentration in aqueous solution was measured using a UV/VIS spectrophotometer (UV-5100, Shanghai Metash Instruments Company), and the following Eq. (14) was used to evaluate the removal efficiency [15, 22]:

14 $$\:\text{R}\text{E}\text{\%}\:=\left[\right(\text{C}\text{o}\:-\:\text{C})\:/\:\text{C}\text{o}]\:\text{*}100$$

Where C_o_ and C are starting and finishing concentrations in Molar for each heavy metal ion, respectively.

### Limitations of this study

One of the experimental limitations occurs during preparation of the (Carbonized Chitosan-Fe_3_O_4_-SiO_2_) nano-composite using co-precipitation method is the difficulty in particle size control and the nano-particles distribution on the surface of the composite. Moreover, using the co-precipitation method may also produce non-uniform particle sizes and distributions, which affects the properties of the nanocomposite. In addition, to achieve the desired distribution and structure, precise conditions will be required which can be challenging to replicate consistently. Using the synthesized nano-composite for adsorption may not be very selective towards particular metal ions, resulting in competitive adsorption behaviors and lower efficiency in the removal and recovery of individual metal ions from a mixture. It can be challenging to completely desorption all the metal ions and regenerate the active sites of the synthesized nano-composite for reuse without losing its adsorptive capacity and performance. The removal of metal ions is sensitive to pH thus; it is necessary to determine the suitable pH before starting the adsorption experiments.

### Experimental design

A wide range of parameters are involved in the process of surface adsorption. As a result, maximizing these parameters is essential to obtaining a high analyte surface adsorption rate. Response Surface Methodology (RSM) – Central Composite Design (CCD) has become more and more well-known as the most effective statistical methodology for examining and maximizing the parameters of diverse processes within the range of protocols and procedures that are available [27]. RSM-CCD uses a variety of statistical and mathematical methods to construct an experimental model with the aim of optimizing response through thoughtful experiment design that concurrently elucidates the interdependent relationships between variables [27]. The first step in this analysis is to plan a number of tests in order to get enough response predictions [28]. The influence of various factors on the accuracy of the result can then be ascertained by fitting a hypothetical (empirical) model to the data acquired in the chosen design and ultimately figuring out the ideal conditions on the model’s input variables, leading to maximizing or minimizing the study’s response [27]. Furthermore, it may determine the mix of parameters and values required to optimum effectiveness (pollutant removal) by looking at parametric effects and interactions. RSM modelling and the Design Expert v.13 program were used in the design and optimization of the reaction parameters. The number of CCD-designed experiments was determined by Eq. (15) [28]:

15 $$\:N={k}^{2}+2k+n$$

Where k is the number of factors examined, n is the number of replicates, and N is the total number of experiments.

The position of the axial points within the experimental domain can be inferred from the value of alpha in CCD, which is an important consideration [28]. The alpha value, which can be either orthogonal or spherical, dictates the design of a CCD. In addition, the design is usually in the middle of being either face centered or rotatable. Consequently, the design is computed using Eq. (16) [28]:

16 $$\:\alpha\:={{(2}^{k})}^{0.25}$$

Because it ensures that the axial point in the factorial portion of the design is in the proper location, alpha = 1 is the desirable value to be used. Three levels of factors need to be included in the design matrix for this kind of design, which is referred to as face centered design [28]. A system’s behavior can be mathematically explained by specifying the relationship between its inputs and outputs [28]. The behavior of the system can be explained by a second-order polynomial model, sometimes known as a quadratic model. This model is represented by Eq. (17) [21, 22]:

17 $$\eqalign{& \>{\rm{Y}} = \cr & \beta {\>_o}\> + \sum \> _{i = 1}^k\beta {\>_{i\>\>}}{x_{i\>}} + \sum \> _{i = 1}^k\beta {\>_{ii\>\>}}{x_{ii\>}}^2 + \cr & \sum {\>_{i = 1}^k} \sum {\>_{j = 1}^k} \beta {\>_{ij}}{x_i}{x_j} + \in}$$

Here, Y represents the responses, k is the total number independent factors, $\:{\beta\:}_{o}$ is an intercept, i, ii, and ij with β represent the coefficient values for linear, quadratic, and interaction effects, respectively, and xi and xj in the above equation show the coded levels for independent variables. For this research, Eq. (17) is written as Eq. (18) [21, 22]:

18 $$\eqalign{& \>{Y_i} = \cr & \beta {\>_o} + \>\beta {\>_1}{x_1} + \>\beta {\>_2}{x_2} + \>\beta {\>_3}{x_3} + \>\beta {\>_{11}}{x_{21}} + \> \cr & \beta {\>_{22}}{x_{22}} + \>\beta {\>_{33}}{x_{23}} + \>\beta {\>_{12}}{x_1}{x_2} + \>\beta {\>_{13}}{x_1}{x_3} + \>\beta {\>_{23}}{x_2}{x_3} \cr}$$

Table 1 illustrated the experimental design matrix generated by (CCD) including three responses of the removal percents of nickel, cobalt and copper metal ions from aqueous solutions. Based on the information shown in Table 1, the Analysis of Variance (ANOVA) and multiple regression analyses in the (CCD) with quadratic model Eq. (18) were carried out.

**Table 1:** Experimental design matrix

Run	A: Initial concentration of heavy metals ions	B: Contact time	C: Adsorbent dose	Percent removal of nickel ion	Percent removal of cobalt ion	Percent removal of copper ion
	(Molar)	(Min)	(g/L)	%	%	%
1	0.1	48	2	38.6	15.39	24.38
2	0.1	90	3.5	66.53	23.73	34.725
3	0.075	48	2	70.82	38.45	55.36
4	0.1	90	0.5	30.57	10.09	18.06
5	0.05	48	2	87.73	57.86	68.49
6	0.075	48	2	70.82	38.45	55.36
7	0.075	6	2	48.67	21.64	35.08
8	0.075	90	2	75.28	45.95	60.17
9	0.05	90	0.5	71.77	48.75	52.706
10	0.1	6	3.5	20.8	5	10.89
11	0.075	48	2	70.82	38.45	55.36
12	0.05	6	0.5	54.93	30.5	42.78
13	0.05	6	3.5	61.11	38.17	50.56
14	0.1	6	0.5	10.63	1.58	4.411
15	0.075	48	2	70.82	38.45	55.36
16	0.075	48	0.5	60.28	28.28	43.27
17	0.075	48	2	70.82	38.45	55.36
18	0.05	90	3.5	94.12	64.28	76.23
19	0.075	48	3.5	82.01	52.08	67.85
20	0.075	48	2	70.82	38.45	55.36

## Results and discussion

### Scanning electron microscopy (SEM)

**Fig. 3:**
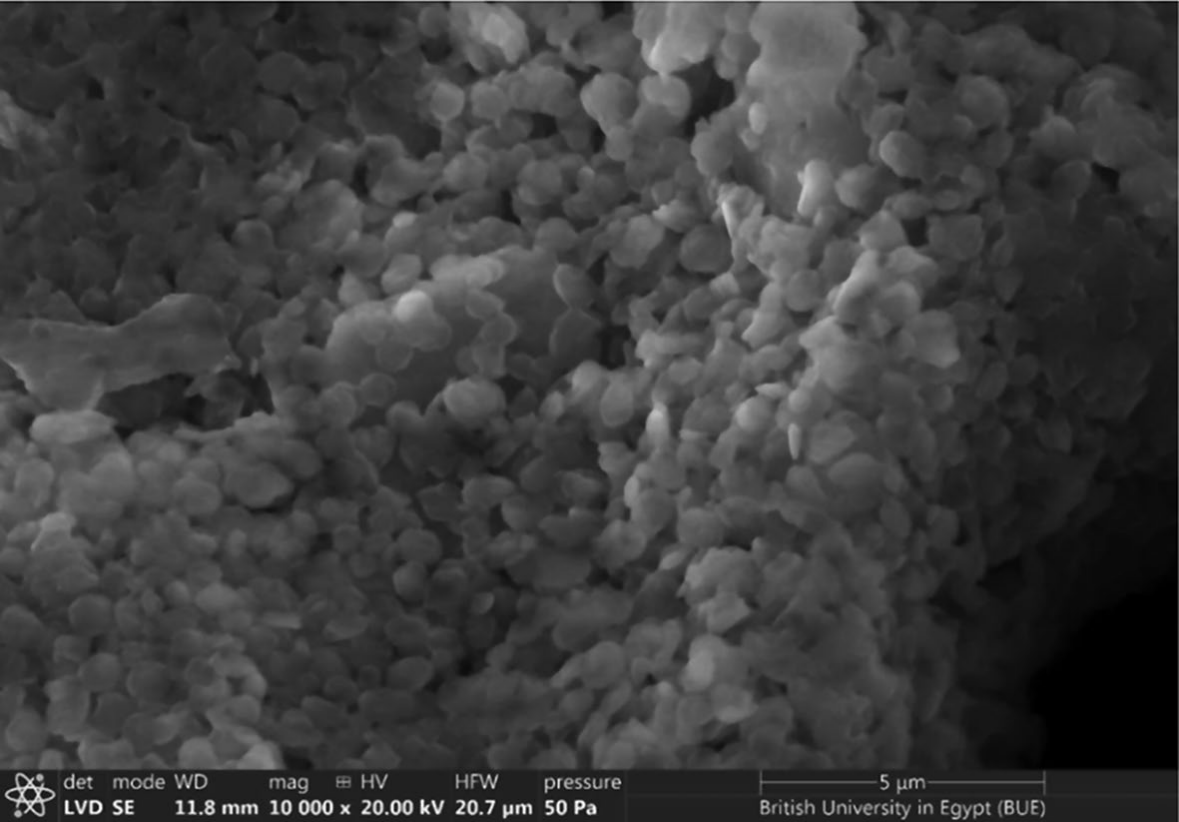
SEM of sodium silicate (Na_2_SiO_3_)

Figure 3 represented the SEM of the extracted Na_2_SiO_3_ from sugarcane bagasse. This figure illustrated a heterogeneous and porous surface with almost equally sized spherical particles.

**Fig. 4:**
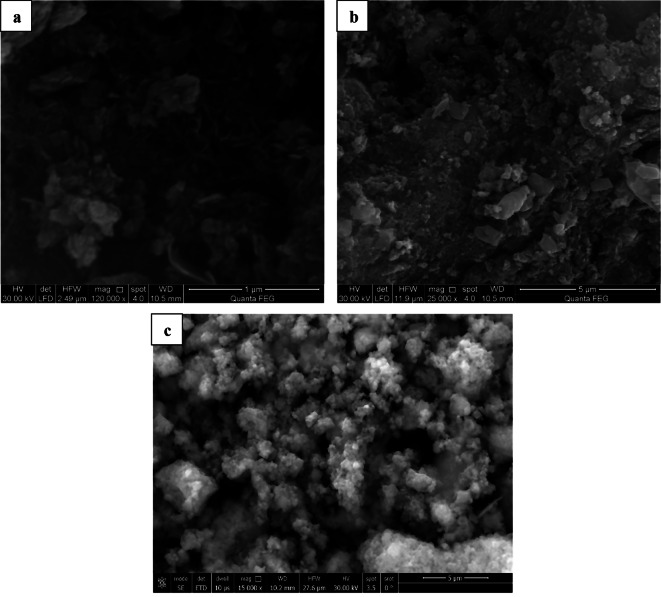
SEM of the Carbonized Chitosan (**a**), Carbonized Chitosan-Fe_3_O_4_ (**b**) and (Carbonized Chitosan-Fe_3_O_4_-SiO_2_) nano-composite (**c**)

Figure 4 depicted the surface morphology of Carbonized Chitosan, Carbonized Chitosan-Fe_3_O_4_ and Carbonized Chitosan-Fe_3_O_4_-SiO_2_ synthesized nano-composite. The honeycomb carbon structure appeared in Fig. 4a which ensured the successful carbonization of chitosan. The Fe_3_O_4_ particles have been adopted on the surface of Carbonized Chitosan as shown in Fig. 4b where they appeared as irregular spherical particles. The particles’ size of Carbonized Chitosan–Fe_3_O_4_ ranged from 45 nm to 85 nm. The SiO_2_ spherical particles have been distributed on the surface of the Carbonized Chtiosan-Fe_3_O_4_ composite as shown in Fig. 4c. In addition, the Carbonized Chitosan-Fe_3_O_4_-SiO_2_ particles were agglomerated due to the magnetic characteristics of Fe_3_O_4_ with particle sizes ranged from 130 nm to 200 nm.

### Transmission electron microscope (TEM)

**Fig. 5:**
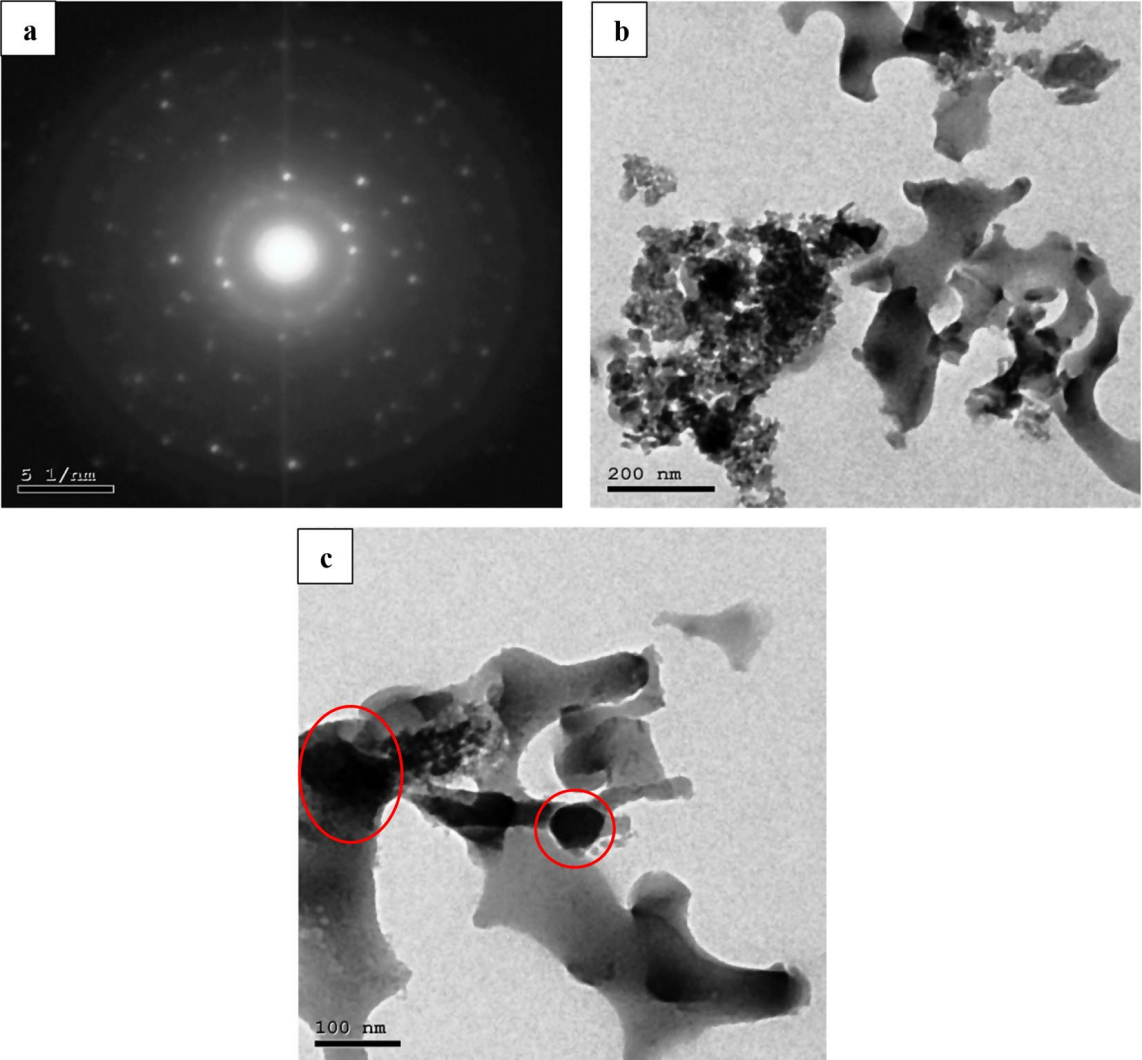
TEM of the synthesized (Carbonized Chitosan-Fe_3_O_4_-SiO_2_) nano-composite (**a**) at 1 nm, (**b**) at 200 nm and 100 nm (**c**)

Figure 5a showed the core/shell structure at 1 nm where the translucent edges were separated from the dark center. This showed that Fe_3_O_4_ first grew on the outermost layer of the Carbonized Chitosan (core), then SiO_2_ grew on the surface of Fe_3_O_4_. Agglomerations appeared in non-spherical structure due to the presence of Fe_3_O_4_ particles as shown in Fig. 5b. Figure 5c detected core/shell structure with nearly spherical shape composed of darker point (Carbonized Chitosan), lighter contrast of Fe_3_O_4_ as the first shell and the lightest contrast of SiO_2_ as the outer shell.

### Brunauer-emmett-teller (BET)

**Fig. 6:**
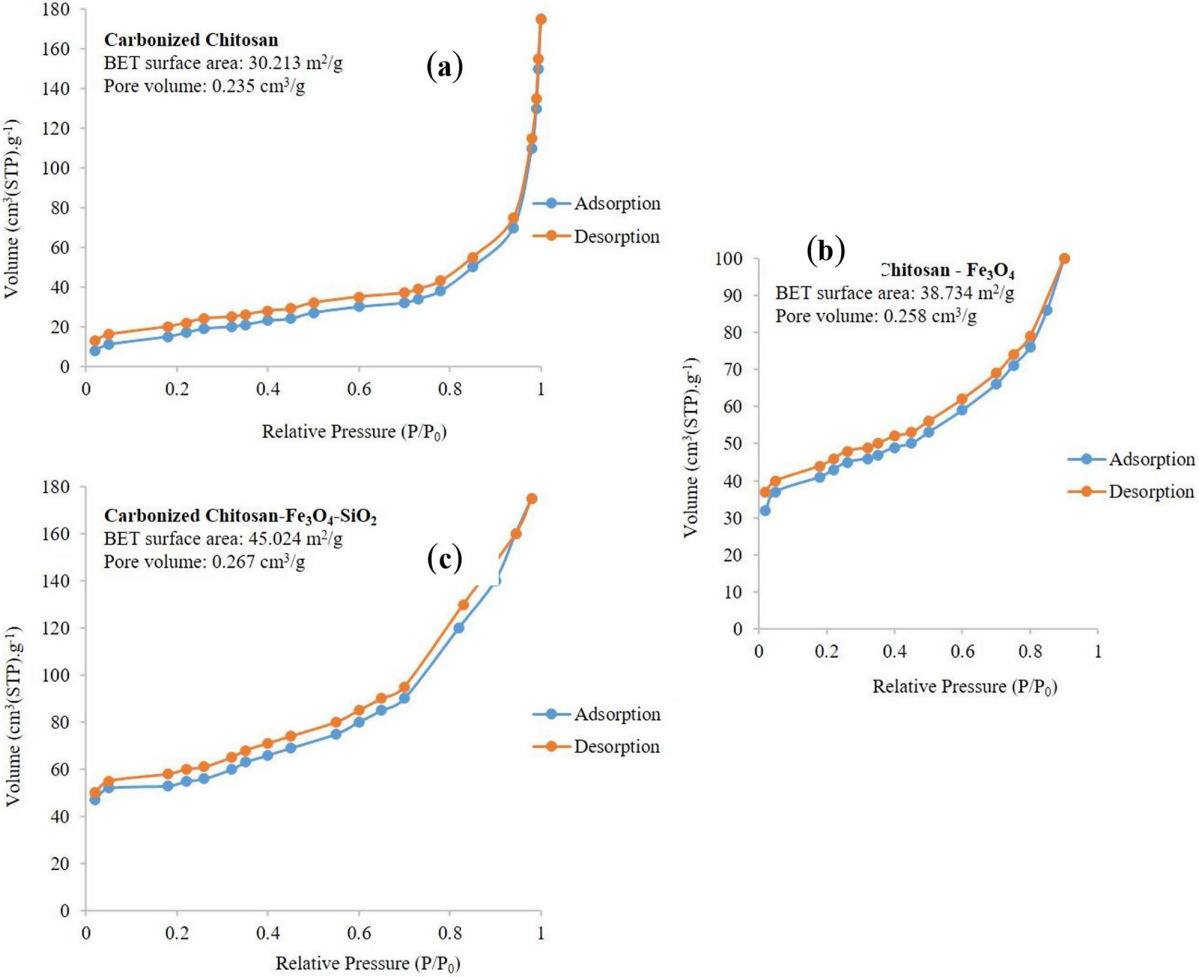
N_2_ adsorption-desorption isotherm for Carbonized Chitosan (**a**), Carbonized Chitosan-Fe_3_O_4_ (**b**) and Carbonized Chitosan-Fe_3_O_4_-SiO_2_ composite (**c**)

BET surface areas, and pore volume of Carbonized Chitosan, Carbonized Chitosan-Fe_3_O_4_ and the synthesized composite Carbonized Chitosan-Fe_3_O_4_-SiO_2_ were studied using N_2_ adsorption (desorption) analysis as represented in Fig. 6. Carbonized Chitosan, Carbonized Chitosan-Fe_3_O_4_ and Carbonized Chitosan-Fe_3_O_4_-SiO_2_ composite had BET surface areas of 30.213 m^2^/g, 38.734 m^2^/g, and 45.024 m^2^/g. In addition, the cumulative BJH pore volumes were 0.235 cm^3^/g, 0.258 cm^3^/g, and 0.267 cm^3^/g, respectively. Based on the BET analysis, Fe_3_O_4_ and SiO_2_ particles provided good contribution in increasing the active surface area of the Carbonized Chitosan.

### X-ray diffraction (XRD)

**Fig. 7:**
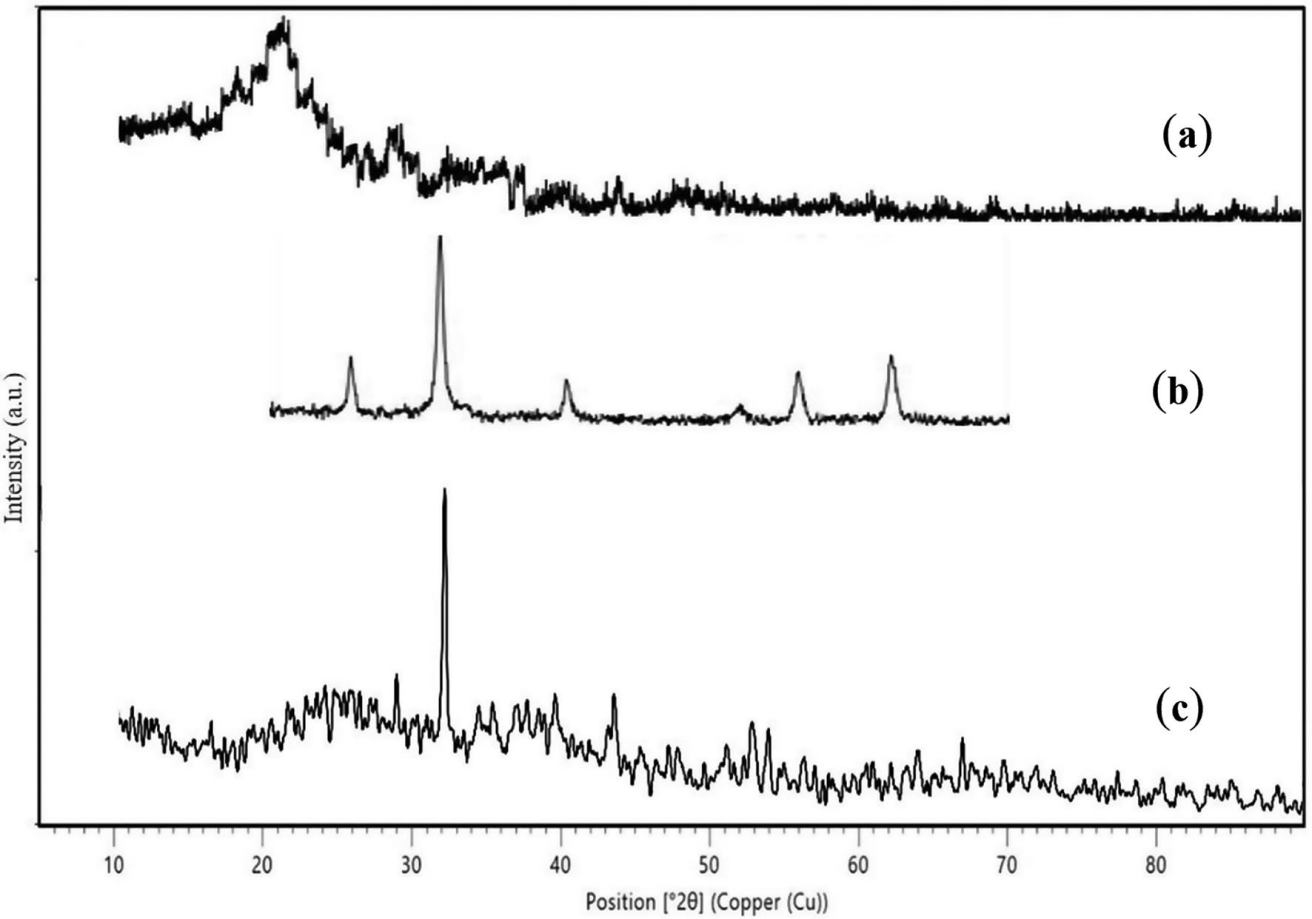
XRD analysis of the Carbonized Chitosan (**a**), Magnetite (**b**), and (Carbonized Chitosan-Fe_3_O_4_-SiO_2_) synthesized nano-composite (**c**)

The XRD patterns of Carbonized Chitosan, Magnetite (Fe_3_O_4_), Silica (SiO_2_) and (Carbonized Chitosan-Fe_3_O_4_-SiO_2_) nano-composite were presented in Fig. 7. Figure 7a showed a broad peak at a range of 2Ɵ = 20-22.53^o^ [29] which indicated the amorphous structure of the Carbonized Chitosan. Figure 7b represented the free magnetite nano-particles with sharp peaks observed at 2Ɵ = 30.23°, 36.36°, 43.23°, 54.25°, 58.35° and 63.10° [30]. After coating of Carbonized Chitosan with Fe_3_O_4_, it was observed an extensive decrease in the peak intensity of the Carbonized Chitosan due to the high crystallinity of magnetite. In addition, SiO_2_ peaks appeared in Fig. 7c at 2Ɵ = 21.8°, 35.97°, 47.67° and 48.55° [31] which ensured the deposition of SiO_2_ particles on the surface of Carbonized Chitosan-Fe_3_O_4_ composite.

### Fourier transformation infrared (FTIR)

**Fig. 8:**
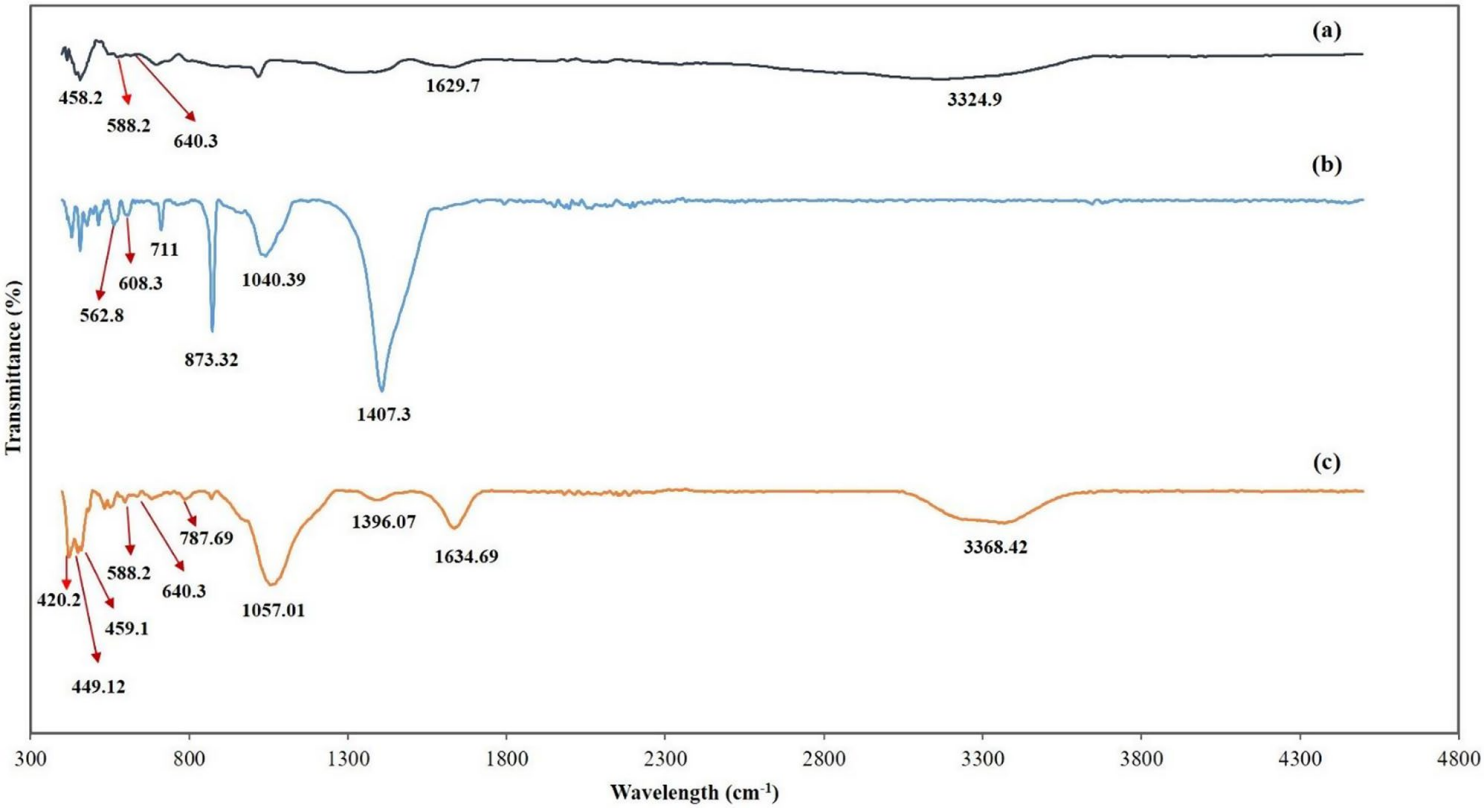
FTIR analysis of free Magnetite (**a**), (Carbonized-Chitosan) (**b**), and (Carbonized Chitosan-Fe_3_O_4_-SiO_2_) synthesized nano-composite (**c**)

Figure 8 represented the FTIR band of free magnetite and (Carbonized Chitosan) versus the (Carbonized Chitosan-Fe_3_O_4_-SiO_2_) synthesized nano-composite. The FTIR analysis was performed under a range of wavelengths between 400 and 4000 cm^− 1^. The observed peaks from 458.2 cm ^to 1^ to 640.3 cm^-1^ at the FTIR analysis of free magnetite revealed to Fe-O bonds in the crystalline lattice of Fe_3_O_4_ [32]. These peaks appeared in the IR of the synthesized nano-composite (Carbonized Chitosan-Fe_3_O_4_-SiO_2_) ensuring the precipitation of Fe_3_O_4_ particles on the surface of the precursor (Carbonized Chitosan). In addition, the peaks at 1629.7 cm^-1^ and 3324.9 cm^-1^ revealed to (-OH) group [32]. Regarding the IR band of the (Carbonized Chitosan), peaks at 873.32 cm^-1^, 711 cm^-1^, 608.3 cm^-1^, 562.8 cm^-1^, 1040.39 cm^-1^ and 1407.3 cm^-1^ revealed to the (C = O) and (C-H) groups. respectively, ensuring the successful carbonization of Chitosan [33]. Peaks in the (Carbonized Chitosan-Fe_3_O_4_-SiO_2_) nano-composite at 1634.69 cm^-1^ and 3368.42 cm^-1^ attributed to (-OH) group [34], these peaks appeared due to washing using distilled water during the preparation of the nano-composite. In addition, two new sharp peaks appeared at 449.12 cm^-1^ and 420.3 cm^-1^ revealed to the (Si-O-Fe) [35]. Moreover, new peaks appeared at 1057.01 cm^-1^ and 787.69 cm^-1^ revealed to (Si-O-Si) and (Si-O) groups, respectively [35]. The disappearance of the IR bands at 711 cm^-1^, 873.32 cm^-1^ and 1040.39 cm^-1^ were due to the co-precipitation of SiO_2_ and Fe_3_O_4_ particles on the surface of the precursor (Carbonized Chitosan).

**Fig. 9:**
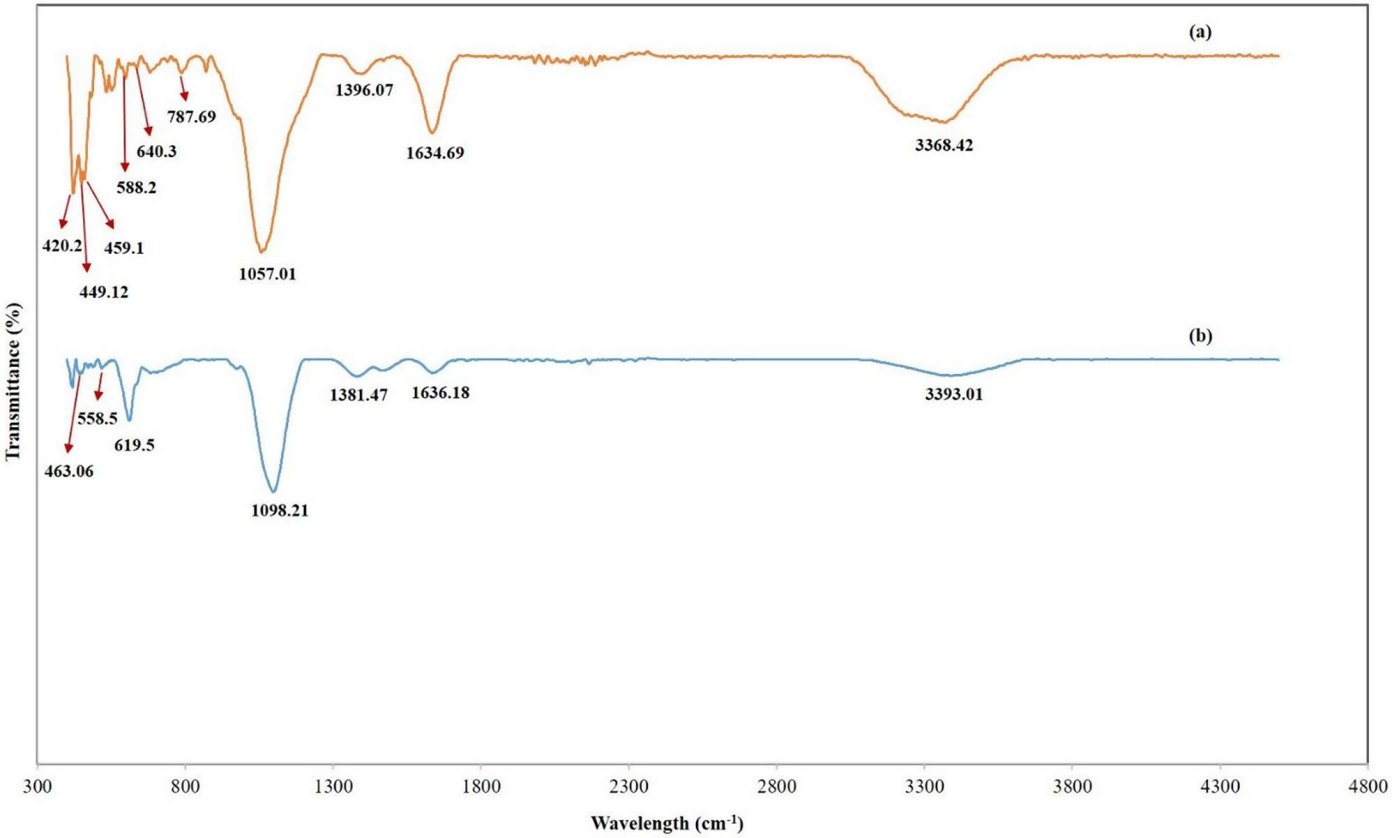
FTIR analysis of the (Carbonized Chitosan-Fe_3_O_4_-SiO_2_) synthesized nano-composite (before) (**a**) and (after) (**b**) simultaneous adsorption process

Figure 9 represented the FTIR band of the FTIR band of the (Carbonized Chitosan-Fe_3_O_4_-SiO_2_) synthesized nano-composite (before) and (after) adsorption of a mixture of heavy metals (Co^2+^, Ni^2+^ and Cu^2+^) from an aqueous solution. A new peak appeared after adsorption at 558.5 cm^-1^ revealed the Co^2+^ ion [36]. Additionally, new peaks appeared after adsorption at 619.5 cm^-1^ and 463.06 attributed to the Cu^2+^ metal ion bounded with C-O group and Ni-O stretching, respectively [33, 36]. The decrease in the intensity of peaks at 3368.42 cm^-1^ and 3393.01 cm^-1^ took place after adsorption indicating different binding between the central metal Co^2+^ ion and ligand [37].

### Zeta potential (ZP)

**Fig. 10:**
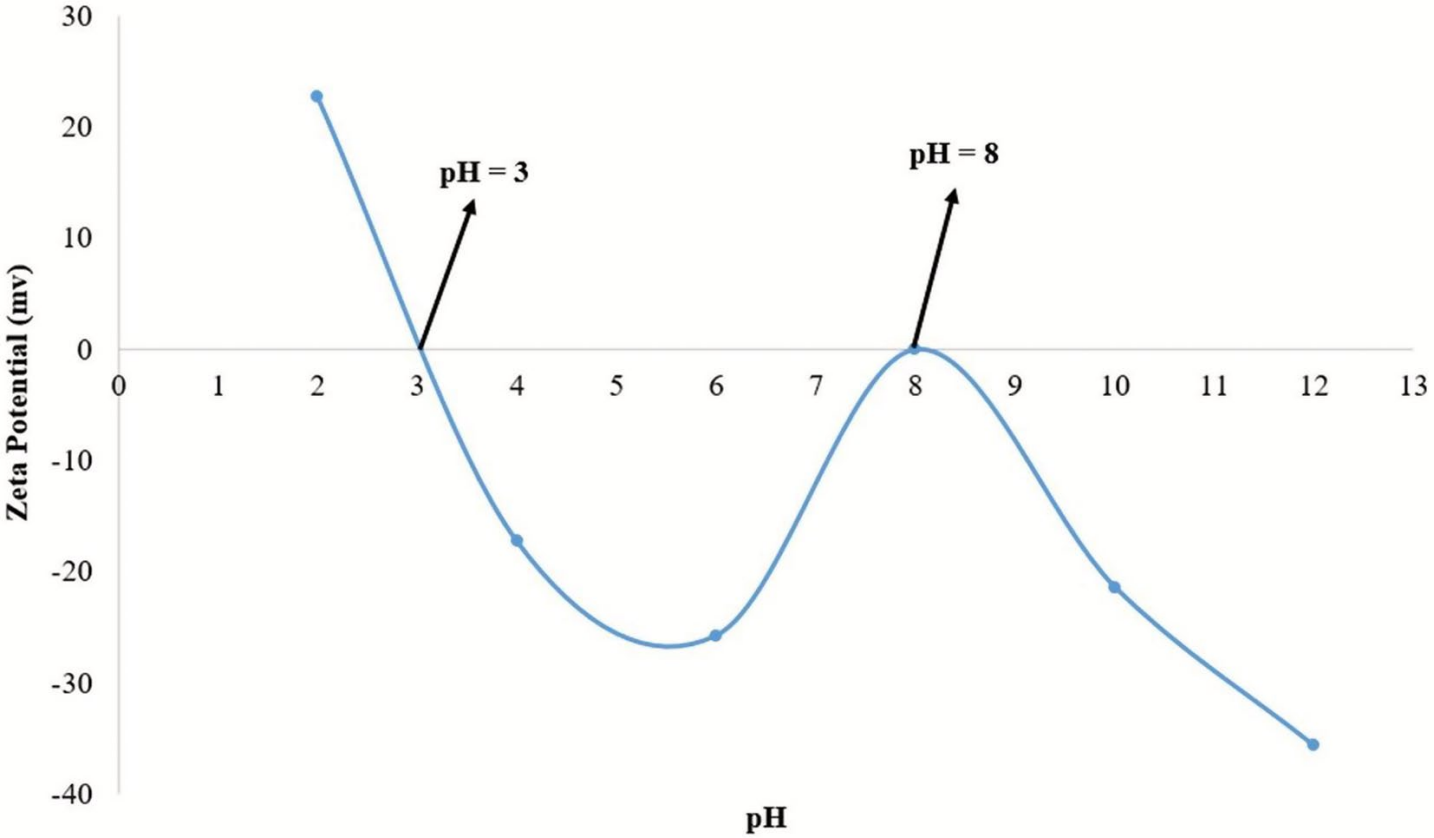
Zeta Potential analysis for the synthesized nano-composite (Carbonized Chitosan-Fe_3_O_4_-SiO_2_)

The difference in potential between the layer of fluid around the surfaces and the bulk fluid containing the particles is measured by the zeta potential in nanoparticle surfaces with oppositely charged ions. The (Carbonized Chitosan-Fe_3_O_4_-SiO_2_) synthetic green magnetic nanocomposite’s isoelectric point must be determined because electrostatic interactions between pollutant loads and particle surfaces influence the adsorption of heavy metal ions (Cu^2+^, Co^2+^, and Ni^2+^) [38]. According to Fig. 10, the synthesized nano-composite (Carbonized Chitosan-Fe_3_O_4_-SiO_2_) exhibited double isoelectric points at pH 3 and pH 8. Double isoelectric points might be resulted from the complex chemistry of Carbonized Chitosan in combination with the other composite components; SiO_2_ and Fe_3_O_4_. Since Carbonized Chitosan and SiO_2_ have theoretical isoelectric points of pH = 3.5 and 3, the lower isoelectric point at pH = 3 was due to their presence [39]. While the higher pH = 8 was due to the presence of Fe_3_O_4_ which had theoretical isoelectric point of 7.9 [37].

### Kinetics study

Pseudo First Order (PFO) and Pseudo Second Order (PSO) kinetics models were used during the kinetics study of this adsorption system. This Kinetics study was performed at different contact times and under the following experimental conditions; pH = 9, initial concentrations of heavy metal ions mixture = 0.1 M, 0.075 M and 0.05 M, temperature = 25 ^o^C and dosage of adsorbent = 2 g/L. As shown in Table 2, the highest R^2^ values were for the PFO model which indicated that the PFO was the best fitted model with the experimental results. Additionally, it was observed that the values of k_1_ in the PFO model with respect to cobalt, nickel and copper ions concentrations of 0.1 M, 0.075 M and 0.05 M were extensively higher than the values of k_2_ for the same metal ion concentrations in the PSO model. Therefore, it could be concluded that the adsorption rate of the heavy metal ions mixture of cobalt, nickel and copper ions from aqueous solutions in the PFO model was higher than the rate in PSO model.

**Table 2:** Results of the PFO and PSO kinetic models

Kinetic model	Initial heavy metals ion concentration (M)	Parameters	with respect to cobalt ion	with respect to nickel ion	with respect to copper ion
PFO	0.1	R^2^	0.9993	0.9955	0.9998
		k_1_ (min^-1^)	0.034	0.059	0.041
	0.075	R^2^	0.9981	0.9976	0.9982
		k_1_ (min^-1^)	0.032	0.036	0.039
	0.05	R^2^	0.9992	0.9997	0.9957
		k_1_ (min^-1^)	0.041	0.065	0.046
PSO	0.1	R^2^	0.1178	0.9896	0.9974
		k_2_ (mg/g.min)	8.67E-06	1.72E-05	2.93E-05
	0.075	R^2^	0.9944	0.9986	0.9985
		k_2_ (mg/g.min)	0.00008	0.00012	0.00011
	0.05	R^2^	0.9978	0.9998	0.9997
		k_2_ (mg/g.min)	0.0001	0.00014	0.00015

### Single-component isotherm study

Langmuir, Freundlich and Dubinin-Radushkevich isotherm models were investigated under fixed experimental conditions of dosage of adsorbent = 3.5 g/L, contact time = 90 min, pH = 9 and at different single nickel, cobalt and copper ions concentrations ranged from 0.05 M to 0.1 M. The adsorption mechanism of this adsorption system could be conducted through determination of the best fitted model with the experimental results. As represented in Table 3, Freundlich isotherm model had higher R^2^ values (0.9876), (0.805) and (0.9694) for nickel, cobalt and copper ions, respectively comparing with the R^2^ values of Langmuir isotherm model. Based on this observation, it was concluded that each single metal ion has been adsorbed from aqueous solutions using the synthesized nano-composite (Carbonized Chitosan-Fe_3_O_4_-SiO_2_) based on a multilayer adsorption mechanism. Additionally, Table 3 illustrated that the synthesized new nano-composite had a high adsorption capacity (q_max_) for the mixture of heavy metal ions of 2000 mg/g, 1666.67 mg/g and 2000 mg/g for nickel, cobalt and copper ions, respectively.

**Table 3:** Isotherm parameters of single nickel, cobalt and copper ions

Isotherm models	Parameters	Nickel	Cobalt	Copper
Langmuir	R^2^	0.9876	0.805	0.9694
	q_max_ (mg/g)	2000	1666.67	2000
	K_L_ (L/mg)	0.0001	0.0096	0.0062
	R_L_ (Separation factor)	0.571	0.017	0.025
Freundlich	R^2^	0.9883	0.8924	0.9868
	1/n	1.302	0.371	0.376
	n	0.768	2.67	2.657
	K_F_ (L/mg)	28.347	139.15	140.67
Dubinin-Radushkevich	B (mol^2^/kJ^2^)	361,984	795.02	1543.2
	E (kJ/mol)	0.001 < 8 kJ/mol	0.025 < 8 kJ/mol	0.018 < 8 kJ/mol
	q_max_ (mg/g)	1289.5	1299.2	1484.6

### Multiple-component isotherm study

Table 4 demonstrated that, in the Modified Langmuir isotherm model, the bonding strength between the ions and the synthesized nano-composite was in the following order: Co^2+^ > Ni^2+^ > Cu^2+^ as the values of K_L_ for cobalt ion = 304.2 (L/mg) > K_L_ value of nickel ion = 117.3 (L/mg) > K_L_ value of copper ion = 78.4 (L/mg). In addition, it was worthy to observe that the maximum adsorption capacity (q_max_) = 2908.92 mg/g in case of multiple-component system was less than the summation of q_max_ for each metal ion = 4761.91 mg/g. The reason may be due to the occurrence of various binding sites on the surface of the prepared nano-composite, each with varying degrees of specificity toward the individual cobalt, nickel, and copper ions, or to the partial overlying of adsorption active sites for cobalt, nickel, and copper ions in the tertiary system. Additionally, it was evident that the Extended Freundlich isotherm model’s values for the Marquardt’s Standard Deviation (MPSD) of the cobalt, nickel, and copper ions were lower than those of the Modified Langmuir isotherm model. Thus, The Extended Freundlich isotherm model was the best matched model with the experimental data indicating that the adsorption mechanism was a multi-layer adsorption for this system.

**Table 4:** Tertiary isotherm parameters of the multiple component models

Isotherm models	Parameters	x_1_	y_1_	z_1_	*n*	K_F_ (L/mg)	MPSD
Extended Freundlich	Nickel ion adsorption	3.07	-0.78	3.03	3.066	159.11	0.1949
	Cobalt ion adsorption	2.67	-0.91	2.65	2.67	111.92	0.1715
	Copper ion adsorption	2.83	-0.87	2.81	2.86	164.58	0.1956
Modified Langmuir	Parameters	Η	K_L_ (L/mg)	q_max_ (mg/g)	MPSD		
	Nickel ion adsorption	0.00074	117.3	800.04	0.1794		
	Cobalt ion adsorption	0.00728	304.2	1548.73	0.8499		
	Copper ion adsorption	0.00139	78.4	560.15	0.9889		

### Regression model equations development

Quadratic model was the best-fitted model with the experimental results. It has been conducted using the Design Expert software program, USA (Version 13). The effects of three experimental factors were investigated including, the initial nickel, cobalt and copper ions concentrations (molar) (M), contact time (min) and dosage of adsorbent (g/L). Three responses were detected experimentally including; the nickel, cobalt and copper ions adsorption percents.

The quadratic model of the elimination percent of nickel ions after removal of insignificant terms to increase the model’s accuracy could be described in the following Eq. (19):

19 $$\eqalign{& \>{Y_1} = \cr & + 71.31 - 20.25\>A + 14.21\>B + 9.64\>C \cr & + 2.2\>AC + 5.25\>BC - 8.87\>{A^2} - 10.06\>{C^2} \cr}$$

The quadratic model for the removal percent of cobalt ions after removal of insignificant terms could be described in the following Eq. (20):

20 $$\:{Y}_{2}=+39.31-18.38\:A+9.59\:B+6.41\:C-6.81\:{B}^{2}$$

The quadratic model for copper ion removal percent after removal of insignificant terms could be described in the following Eq. (21):

21 $$\eqalign{& \>{Y_3} = \cr & + 55.74 - 19.83\>A + 9.82\>B + \cr & 7.90\>C + 3.24\>BC - 9.87\>{A^2} - 8.68\>{B^2} \cr}$$

Where Y_1_, Y_2_ and Y_3_ represented the nickel ion, cobalt ion and copper ion adsorption percentages, respectively. A, B and C are the initial nickel, cobalt and copper ions concentrations (molar), contact time (min) and dosage of adsorbent (g/L), respectively.

#### Nickel ion adsorption model – effect of process variables

##### A) Effect of initial (Co^2+^, Ni^2+^ and Cu^2+^) ions concentration

**Fig. 11:**
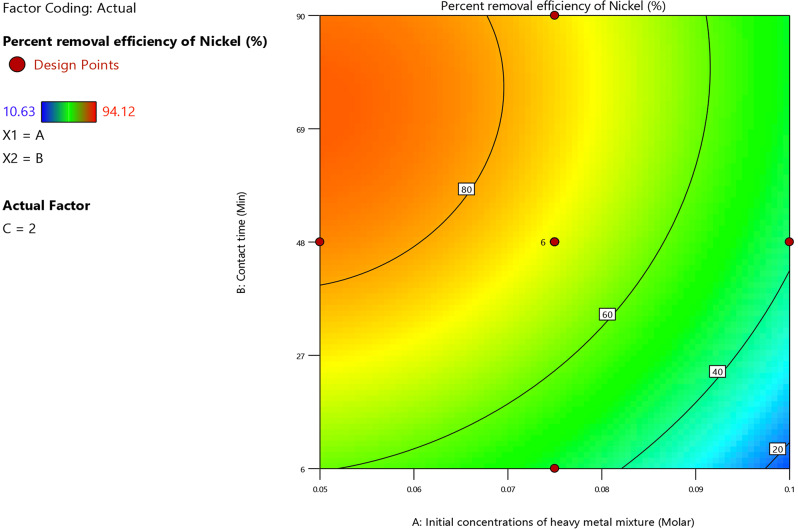
Contour plot of initial metal ions concentration and contact time versus the removal percent of the nickel ion

The initial (Co^2+^, Ni^2+^, and Cu^2+^) ion concentration’s coefficient in the regression Eq. (19) denoted an inverse relationship with the nickel ion adsorption percent. This was brought on by the fact that as the concentration of (Co^2+^, Ni^2+^, and Cu^2+^) ions raised, there were fewer active sites on the adsorbent. Figure 11 depicted that the adsorption percent of nickel ions decreased from 80 to 20% as the initial concentration of the heavy metal ions increased from 0.05 M to 0.098 M at various contact time levels and under the specified experimental conditions of pH = 9, dosage of adsorbent = 2 g/L.

##### B) Effect of the dosage of adsorbent

**Fig. 12:**
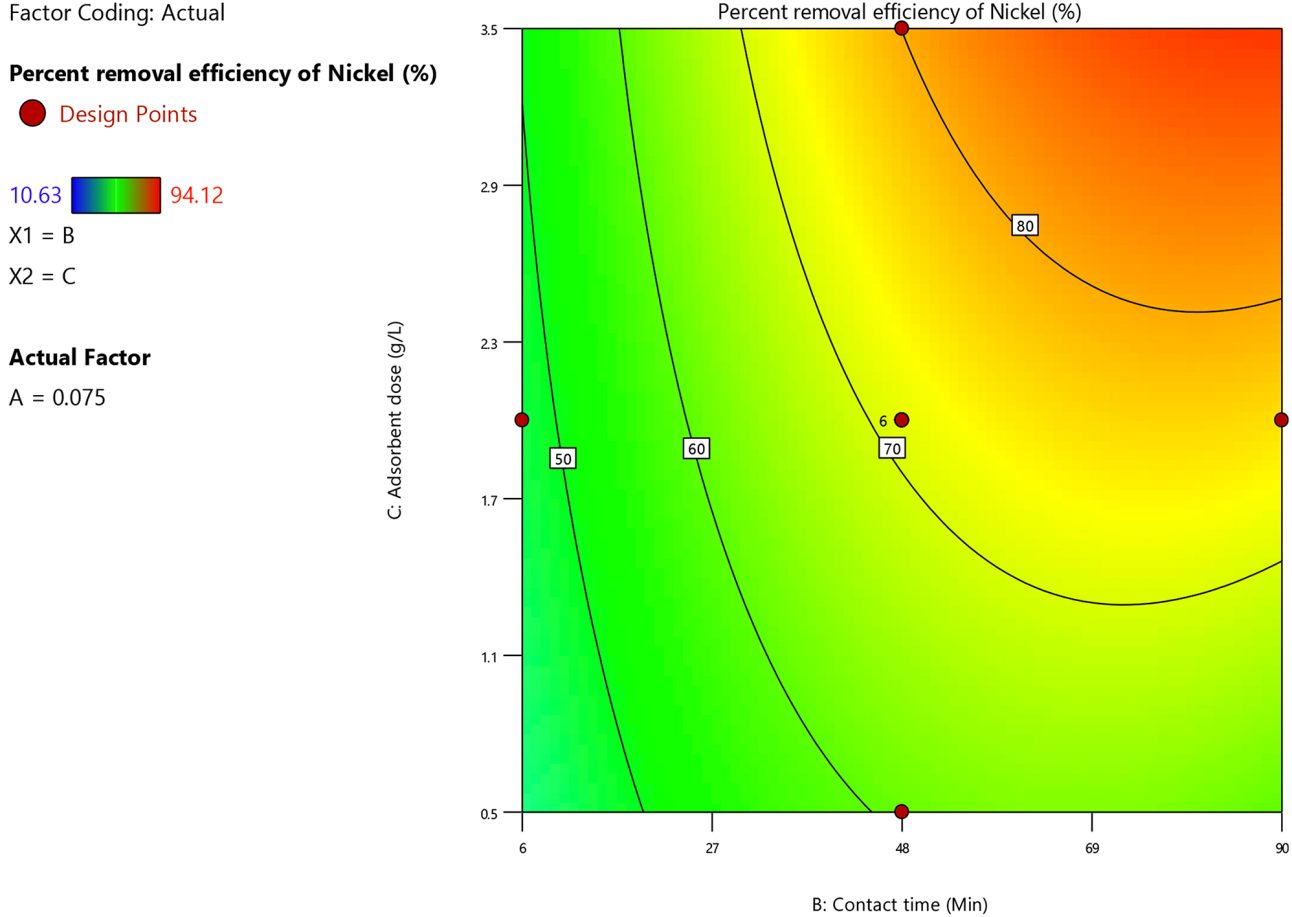
Contour plot of dosage of adsorbent and contact time versus the removal percent of the nickel ion

Dosage of adsorbent and nickel ion adsorption percents were directly proportional, according to the regression Eq. (19). Adsorbent specific surface area increased along with an increase in adsorbent dosage, increasing the amount of available active sites for nickel ion adsorption. Figure 12 depicted that an increase in the dosage of adsorbent from 0.5 to 3.5 g/L had a favourable impact on the nickel ion removal percent as it climbed from 50 to 80% at various contact times and under these specified experimental conditions; pH = 9, initial (Co^2+^, Ni^2+^ and Cu^2+^) ions concentration = 0.075 M.

### Cobalt ion adsorption model – effect of process variables

#### A) Effect of initial (Co^2+^, Ni^2+^ and Cu^2+^) ions concentration

**Fig. 13:**
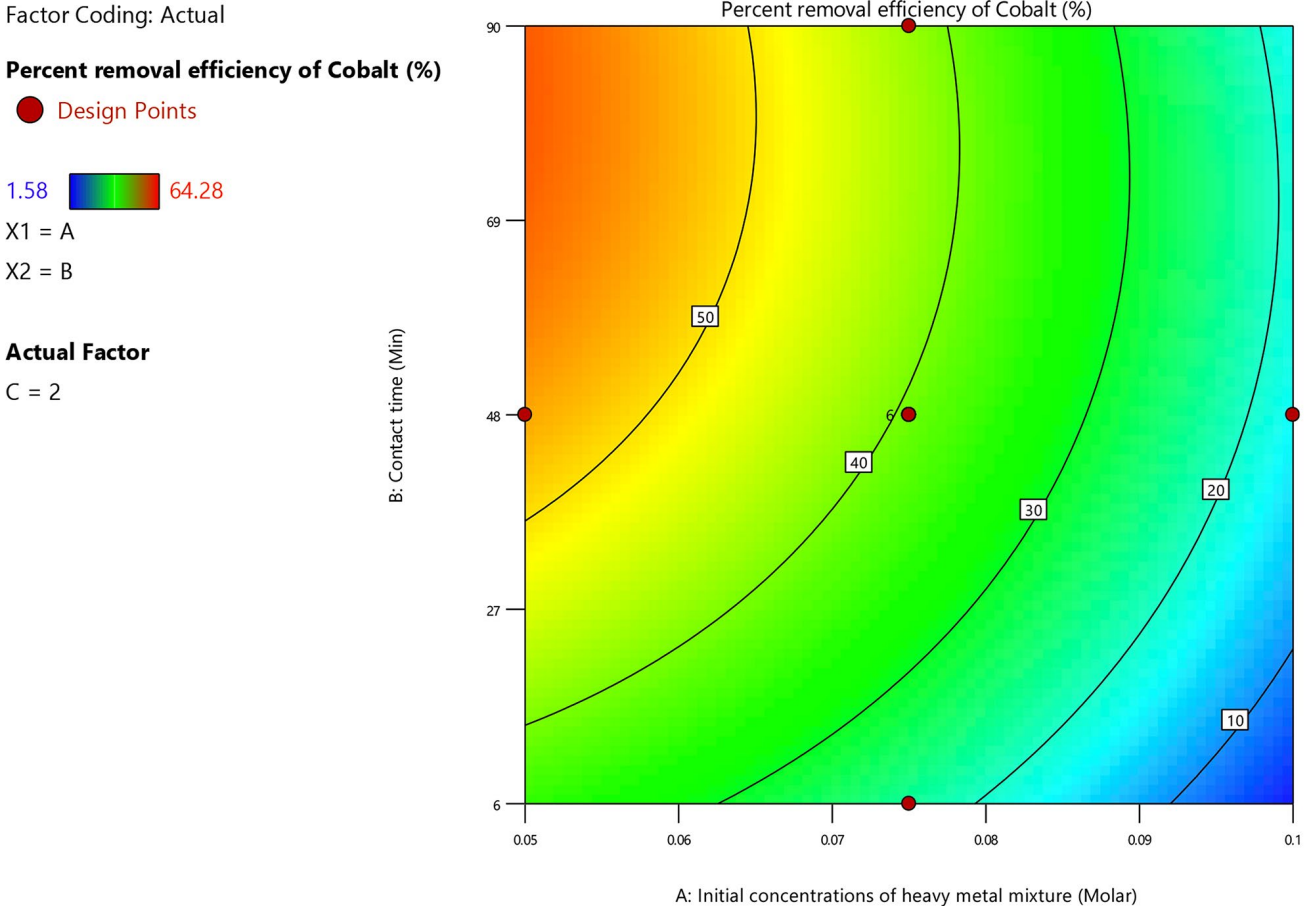
Contour plot for initial (Co^2+^, Ni^2+^ and Cu^2+^) ions concentration and contact time versus the cobalt ion removal percent

The coefficient of the initial (Co^2+^, Ni^2+^ and Cu^2+^) ions concentration in the regression Eq. (20) indicated that there was an inverse proportionality between it and the cobalt ion adsorption percent. This was due to the reduction in active locations on the adsorbent as the (Co^2+^, Ni^2+^ and Cu^2+^) ions concentration increased. According to Fig. 13, cobalt ion adsorption percent decreased from 50 to 10% with a rise in the initial concentration of (Co^2+^, Ni^2+^ and Cu^2+^) ions from 0.05 M to 0.092 M at various levels of contact times and under specified experimental conditions; pH = 9 and dosage of adsorbent = 2 g/L.

#### B) effect of the dosage of adsorbent

**Fig. 14:**
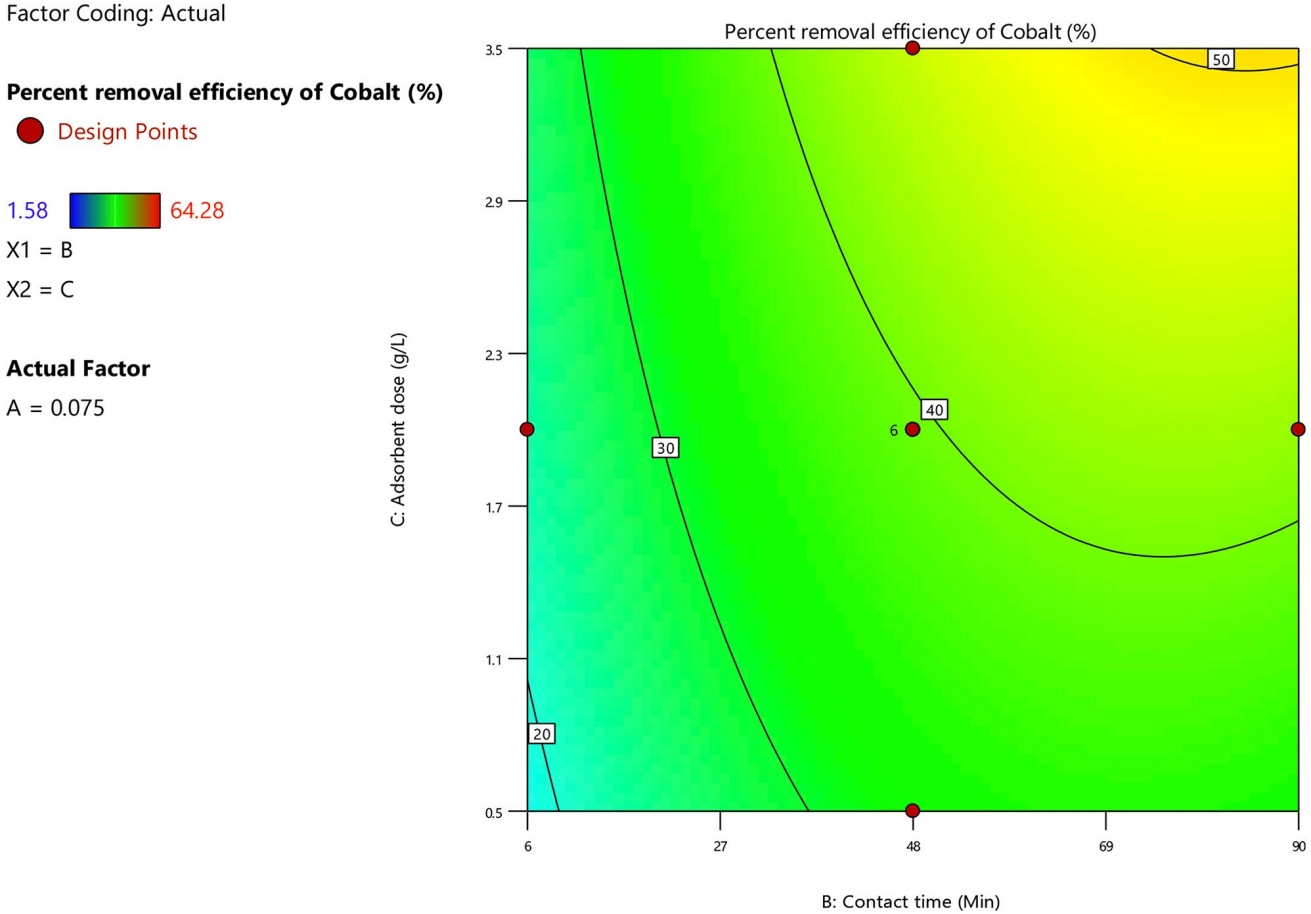
Contour plot for dosage of adsorbent and contact time versus the removal percent of cobalt ion

Referring to the regression Eq. (20), the dosage of adsorbent and the cobalt ion adsorption percent were directly proportional. The exact surface area of the adsorbent increased as the dosage of the adsorbent increased simultaneously, which caused a peak in the availability of active locations for cobalt ion adsorption. The high significant effect of the dosage of adsorbent on the cobalt ion removal percent illustrated in Fig. 14. As an increase in the dosage of adsorbent from 0.5 g/L to 3.5 g/L had a positive effect on the Co^2+^ ion removal percent as it increased from 20 to 50% at various stages of contact times and under specified experimental conditions; pH = 9, initial metal ions concentration = 0.075 M.

### Copper ion adsorption model – effect of process variables

#### A) Effect of initial (Co^2+^, Ni^2+^ and Cu^2+^) ions concentration

**Fig. 15:**
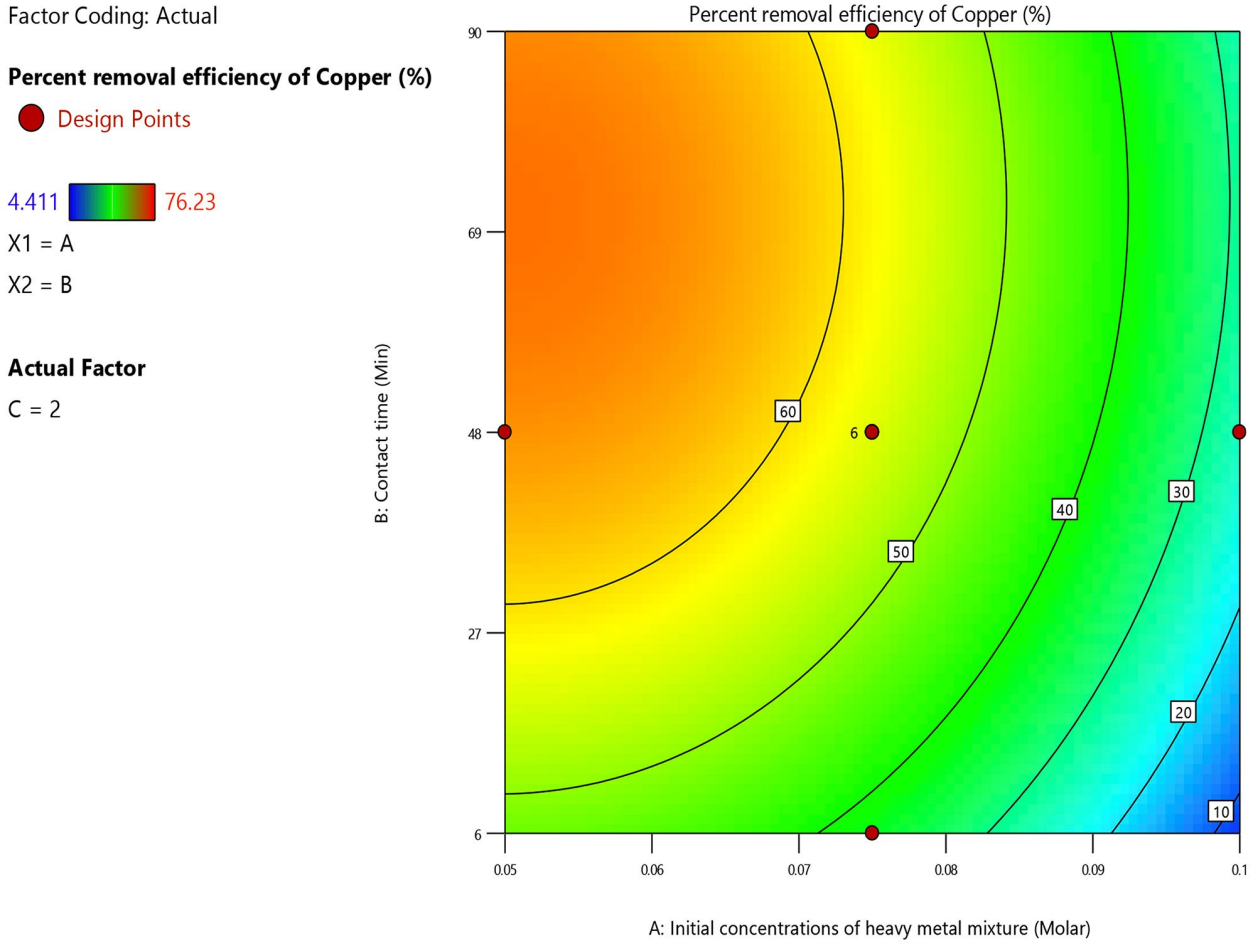
Contour plot of initial metal ions concentration and contact time versus the copper ion removal percent

In the regression Eq. (21), the initial heavy metal ions concentration had a negative sign. This coefficient of the initial metal ions concentration in the regression Eq. (21) indicated that there was an inverse proportionality between it and the copper ion adsorption percent. This was due to the decrease in active sites on the adsorbent as the (Co^2+^, Ni^2+^ and Cu^2+^) ions concentration increased. According to Fig. 15, copper ion adsorption percent decreased from 60 to 10% with a rise in the initial concentration of (Co^2+^, Ni^2+^ and Cu^2+^) ions from 0.05 M to 0.098 M at different levels of contact time and under specified experimental conditions: pH = 9 and dosage of adsorbent = 2 g/L.

#### B) Effect of the dosage of adsorbent

**Fig. 16:**
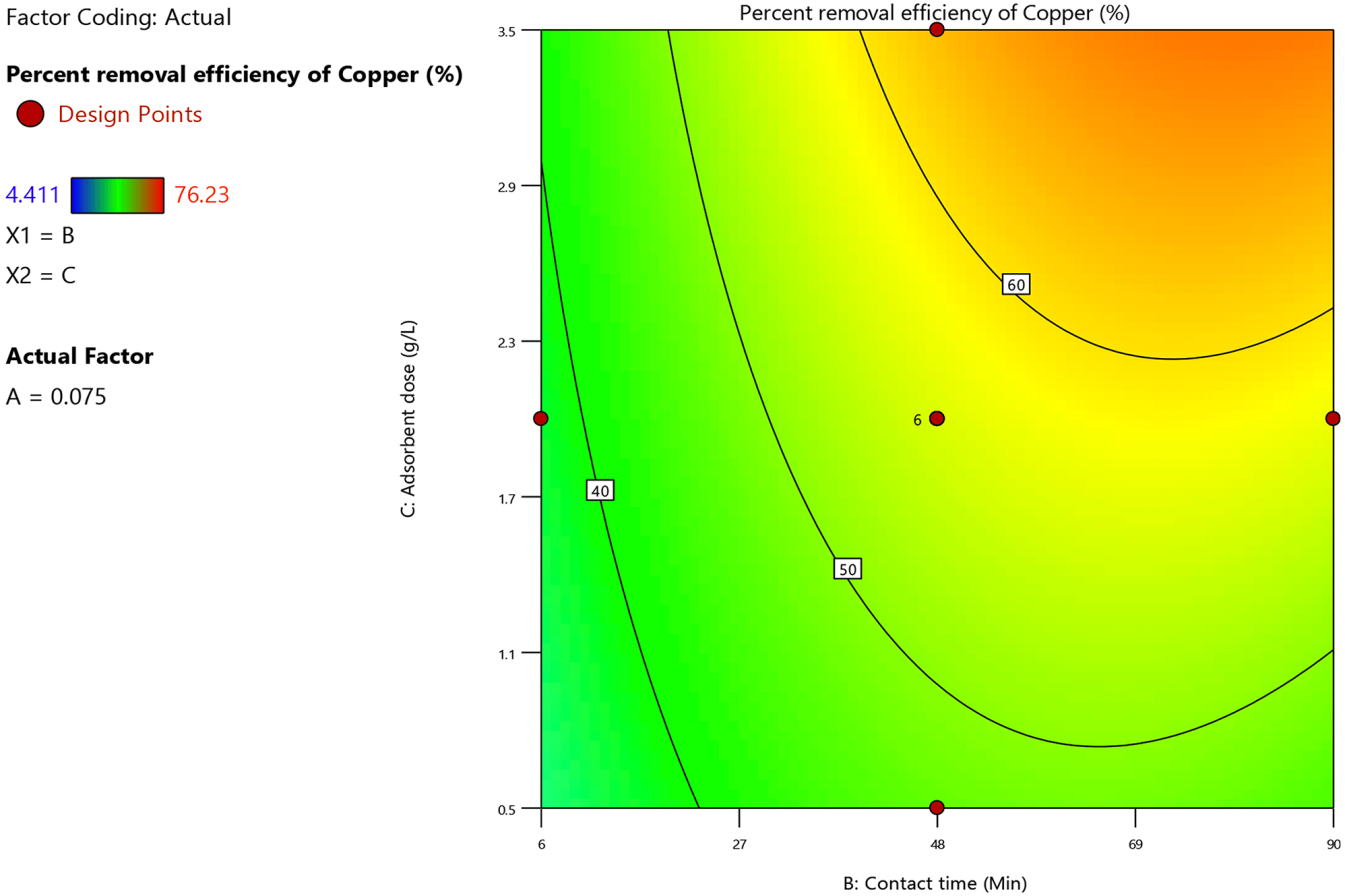
Contour plot of dosage of adsorbent and contact time versus the copper ion removal percent

Based on the regression Eq. (21), the dosage of adsorbent and the copper ion adsorption percent were directly proportional. When the dosage of adsorbent increased, the specific surface area of the adsorbent increased simultaneously which led to an increase in the availability of active sites for copper ion adsorption. The highly significant effect of the dosage of adsorbent on the copper ion removal percent illustrated in Fig. 16. As an increase in the dosage of adsorbent from 0.5 g/L to 3.5 g/L had a positive effect on the Cu^2+^ ion removal percent as it increased from 40 to 60% at different levels of contact time and under these fixed experimental conditions; pH = 9, initial heavy metal ions concentration = 0.075 M.

## Optimization

Numerical optimization was performed to reach the optimum conditions at which the maximum removal percents of Ni^2+^, Co^2+^, and Cu^2+^ metal ions were achieved using Design Expert software. The optimization goals were chosen to reach the maximum removal percents of Ni^2+^, Co^2+^, and Cu^2+^ ions as represented in Table 5.

**Table 5:** Optimization constraints

Factor	Goal	Lower limit	Upper limit	Value
Initial concentration of the heavy metal ions mixture (M)	Minimize	0.05	0.1	---
Contact time (min)	Maximize	6	90	---
Dosage of adsorbent (g/L)	Target	0.5	3.5	2.5
% Nickel ion removal	Maximize	10.63	94.12	---
% Cobalt ion removal	Maximize	1.58	64.28	---
% Copper ion removal	Maximize	4.41	76.23	---

**Fig. 17:**
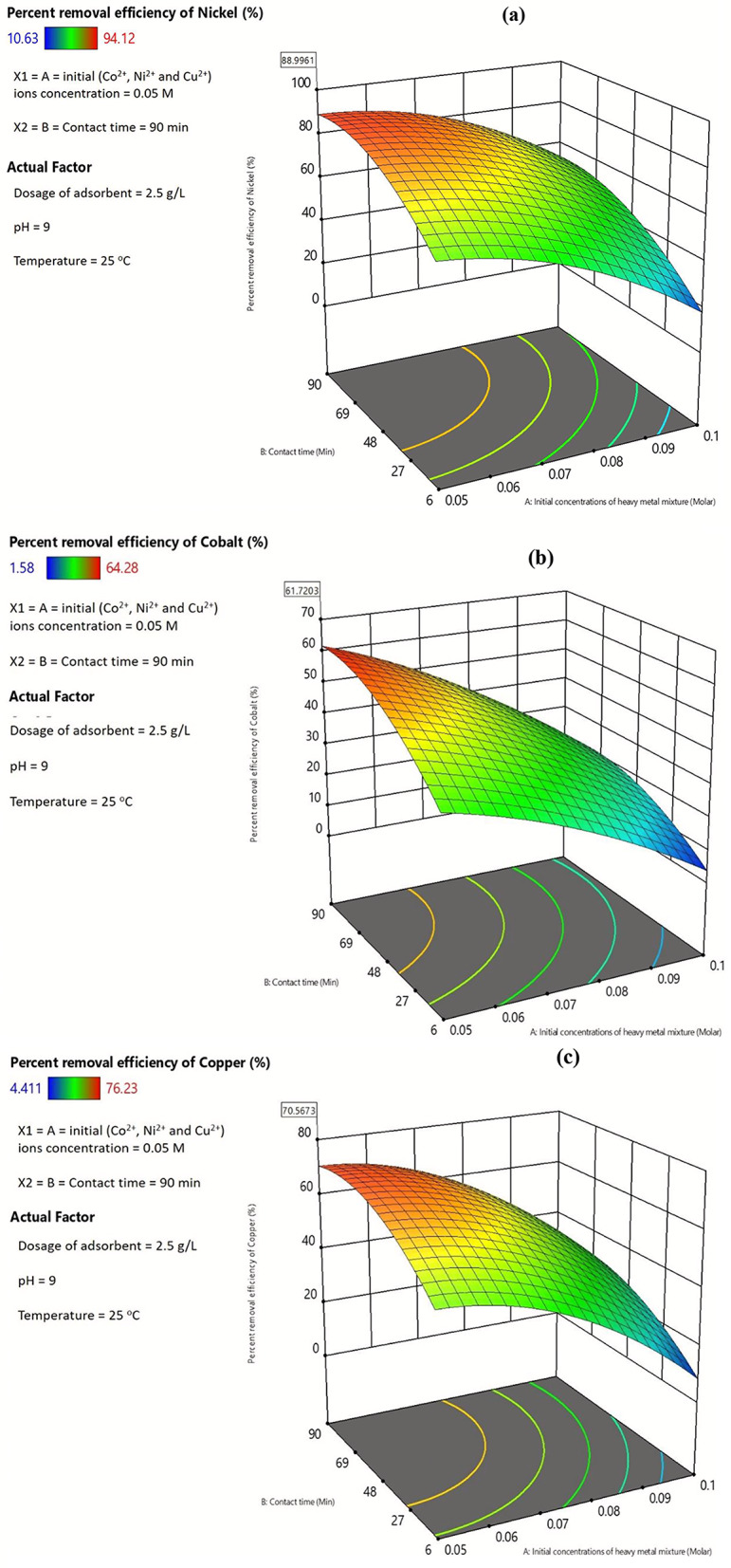
Maximum removal percents of nickel (**a**), cobalt (**b**) and copper (**c**) metal ions from aqueous solutions

Figure 17 a, b and c represented the maximum removal percents of Ni^2+^, Co^2+^, and Cu^2+^ metal ions were 88.99%, 61.72% and 70.56%, respectively. These values were achieved at specified experimental conditions of pH = 9, initial (Co^2+^, Ni^2+^ and Cu^2+^) ions concentration = 0.05 M, dosage of adsorbent = 2.5 g/L, contact time = 90 min and temperature = 25 ^o^C.

## Desorption and regeneration of the spent (Carbonized Chitosan-Fe_3_O_4_-SiO_2_) nano-composite

For desorption of heavy metal ions from wastewater or aqueous solutions, Ethylene Diamine Tetra Acetic Acid (EDTA) solutions or acids such as Nitric acid (HNO_3_) or Hydrochloric acid (HCl) could be used [40]. Five samples of (Carbonized Chitosan-Fe_3_O_4_-SiO_2_) each with 0.1 g were added in conical flasks with 80 mL of 0.05 M of heavy metal ion mixture (Ni^2+^, Co^2+^, and Cu^2+^) at pH = 9. These conical flasks were shaken vigorously at 200 rpm for 90 min at 25 ^o^C followed by filtration of the composite, washing with double distilled water then drying at 40 ^o^C for 7 h. 1 M of the desorption solution HNO_3_ was added to each dried composite after adsorption in conical flasks which were shaken at 200 rpm for 10 min at 25 ^o^C followed by composite filtration, washing with deionized water and drying. the following Eq. (22) was used to evaluate the desorption efficiency [40]:

22 $${\rm{Desorption}}\>{\rm{efficiency}}\>\left( {{\eta _{des}}} \right) = \left( {{{{{\rm{C}}_{{\rm{desorption}}}}} \over {{{\rm{C}}_{{\rm{adsorption}}}}}}} \right) \times \>100$$

C_adsorption_ represents the equilibrium concentration of heavy metal ions on the surface of adsorbent and C_desorption_ represents the concentration of heavy metal ions in the desorption solution.

The five samples of (Carbonized Chitosan-Fe_3_O_4_-SiO_2_) composite from adsorption were firstly washed with de-ionized water then dried in an oven for 40 ^o^C for 4 h. The dried composite samples with dosage of (2.5 g/L) were added to heavy metal ions solution with concentration of 0.05 M, pH = 9, temp = 25 ^o^C, and contact time = 90 min. Five cycles of (Carbonized Chitosan-Fe_3_O_4_-SiO_2_) reusability were conducted using the same experimental conditions mentioned above. The reusability efficiency for each cycle was determined using the following Eq. (23) [40]:

23 $$\:\text{R}\text{e}\text{u}\text{s}\text{a}\text{b}\text{i}\text{l}\text{i}\text{t}\text{y}\:\text{e}\text{f}\text{f}\text{i}\text{c}\text{i}\text{e}\text{n}\text{c}\text{y}=\left(\frac{{\text{C}}_{\text{a}\text{d}\text{s}\text{o}\text{r}\text{p}\text{t}\text{i}\text{o}\text{n}}}{{\text{C}}_{\text{o}}}\right)\times\:100$$

C_adsorption_ represents the equilibrium concentration of heavy metal ions on the surface of adsorbent and C_o_ represents the initial concentration of heavy metal ions in the synthetic aqueous solution.

All metal ions studied were successfully desorbed at 100% efficiency. According to these results, it was possible to recover and reuse the (Carbonized Chitosan-Fe_3_O_4_-SiO_2_) synthesized nano-adsorbent without losing its adsorption capacity, as well as the metal ions. Using the (Carbonized Chitosan-Fe_3_O_4_-SiO_2_) adsorbent, the potential of recovering metal ions (Co^2+^, Cu^2+^, and Ni^2+^) as well as the adsorbent, would certainly improve the economic feasibility of the adsorption process.

## Adsorption of Co^2+^, Cu^2+^, and Ni^2+^ using the synthesized nano-composite versus different adsorbents

The results of the present study were compared with the results of similar studies in terms of absorption capacity, operating conditions and adsorbent regeneration. Table 6 displayed a variety of adsorbent materials that were previously documented for their ability to eliminate Co^2+^, Cu^2+^, and Ni^2+^ from aqueous solutions in batch mode. As represented in Table 6, the prepared (Carbonized Chitosan-Fe_3_O_4_-SiO_2_) nano-composite could be considered as one of the most efficient adsorbents recently synthesized for the purpose of adsorbing Co^2+^, Cu^2+^, and Ni^2+^, when compared to other previously reported adsorbents.

**Table 6:** Comparison of adsorption capacity for the removal of Co^2+^, Cu^2+^, and Ni^2+^ metal ions by different adsorbents

Adsorbent	pH	Contact time	Initial Conc. (Molar)	Maximum adsorption capacity (q_max_) (mg/g)			Temp (^o^C)	Ref.
				Ni^2+^	Co^2+^	Cu^2+^		
Chitosan/clinoptilolite	5	24 h	0.07 for each metal ion	247.03	467.90	719.3	25	[41]
Chitosan/methacrylic acid nanoparticles	6	5 h	0.01 each	340	220	195	20	[2]
Carboxylate-functionalized sugarcane bagasse (SPA)	5.5–5.75	3 h	0.05 each	54.7	33.04	59.42	25	[1]
Poly[N-(4-[4-(amino phenyl) methyl phenyl methacrylamide])] (PAMMAm)	5.5-6	30 min	0.017 each	110.92	108.96	66.09	25	[42]
Hazelnut husks carbon activated with phosphoric acid (HHPAAC)	6	24 h	0.06 each	16.3	17.3	24.3	25	[43]
Carbonized Chitosan-Fe_3_O_4_-SiO_2_	9	90 min	0.1 each	800.04	1548.7	78.4	25	This study

## Future work

Scale-up and commercialization: Once the process has been optimized, there is potential for scale-up and commercialization of the technology. This will involve working with industry partners to develop large-scale systems for removal of a mixture of heavy metal ions from wastewater using green magnetic nano-composite (Carbonized Chitosan-Fe_3_O_4_-SiO_2_).

Environmental impact assessment: the environmental impact of this process will be explored including the energy required for it and the potential for waste disposal issues. This will help to ensure that the process is sustainable and environmentally friendly.

## Conclusions

A new green magnetic nano-composite (Carbonized Chitosan-Fe_3_O_4_-SiO_2_) was synthesized using co-precipitation method. Surface characterization of the synthesized nano-composite was performed including SEM, TEM, BET, XRD, FTIR, and ZP and, to ensure that the preparation of the new green nano-composite was achieved successfully. The kinetics models of this adsorption system were studied where the best fitted model with the experimental results was Pseudo First Order (PFO) model. Dubinin-Radushkevich was studied for single-component adsorption system indicated that the type of adsorption was physical adsorption. Extended Freundlich multiple-component isotherm was the best-fitted model indicating that the adsorption mechanism was a multi-layer adsorption for this system. Regarding the multiple-component system, the maximum adsorption capacity of Ni^2+^, Co^2+^ and Cu^2+^ were 800.04 mg/g, 1548.73 mg/g and 560.15 mg/g, respectively. In this study, a wide range of experimental parameters were investigated, including the initial concentrations of the heavy metals mixture of nickel, cobalt and copper ions (0.05, 0.075, and 0.1 M), the contact time (6, 48, and 90 min), as well as the dosage of adsorbent (0.5, 2, and 3.5 g/L) to assess their impact on the adsorption percents of the heavy metals mixture of nickel, cobalt and copper ions. The optimum adsorption percents of nickel, cobalt and copper ions of 88.99%, 61.72%, and 70.56%, respectively were achieved at specified experimental conditions of pH = 9, initial (Co^2+^, Ni^2+^ and Cu^2+^) ions concentration = 0.05 M, dosage of adsorbent = 2.5 g/L, and contact time = 90 min.

## Data Availability

The data used to support this study’s findings are available from the corresponding author upon request.
